# Impact of Catalysis-Relevant Oxidation and Annealing Treatments on Nanostructured GaRh Alloys

**DOI:** 10.1021/acsami.4c02286

**Published:** 2024-04-09

**Authors:** Tzung-En Hsieh, Johannes Frisch, Regan G. Wilks, Christian Papp, Marcus Bär

**Affiliations:** † Department Interface Design, Helmholtz-Zentrum Berlin für Materialien und Energie GmbH (HZB), 12489 Berlin, Germany; ‡ Department of Chemistry and Pharmacy, Friedrich-Alexander-Universität Erlangen-Nürnberg (FAU), 91058 Erlangen, Germany; § Energy Materials In-situ Laboratory Berlin (EMIL), HZB, 12489 Berlin, Germany; ∥ Department X-ray Spectroscopy at Interfaces of Thin Films, Helmholtz-Institute Erlangen-Nürnberg for Renewable Energy (HI ERN), 12489 Berlin, Germany; ⊥ Freie Universität Berlin, Physical and Theoretical Chemistry, 14195Berlin, Germany

**Keywords:** gallium, rhodium, liquid metals, photoelectron spectroscopy, SCALMS, oxidation, reduction, surface structure

## Abstract

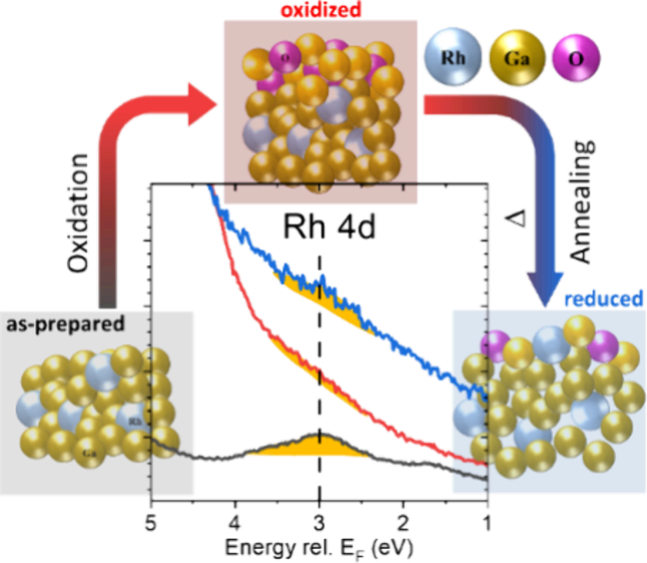

In this study, we examine the surface-derived electronic and chemical structures of nanostructured GaRh alloys as a model system for supported catalytically active liquid metal solutions (SCALMS), a novel catalyst candidate for dehydrogenation reactions that are important for the petrochemical and hydrogen energy industry. It is reported that under ambient conditions, SCALMS tends to form a gallium oxide shell, which can be removed by an activation treatment at elevated temperatures and hydrogen flow to enhance the catalytic reactivity. We prepared a 7 at. % Rh containing the GaRh sample and interrogated the evolution of the surface chemical and electronic structure by photoelectron spectroscopy (complemented by scanning electron microscopy) upon performing surface oxidation and (activation treatment mimicking) annealing treatments in ultrahigh vacuum conditions. The initially pronounced Rh 4d and Fermi level-derived states in the valence band spectra disappear upon oxidation (due to formation of a GaO_*x*_ shell) but reemerge upon annealing, especially for temperatures of 600 °C and above, i.e., when the GaO_*x*_ shell is efficiently being removed and the Ga matrix is expected to be liquid. At the same temperature, new spectroscopic features at both the high and low binding energy sides of the Rh 3d_5/2_ spectra are observed, which we attribute to new GaRh species with depleted and enriched Rh contents, respectively. A liquefied and GaO_*x*_-free surface is also expected for GaRh SCALMS at reaction conditions, and thus the revealed high-temperature properties of the GaRh alloy provide insights about respective catalysts at work.

## Introduction

Efficient conversion of light alkanes into alkenes via nonoxidative dehydrogenation is a long-termed significant research topic due to growing demands in petrochemical industry, e.g., for polymer production or for liquid organic hydrogen carrier (LOHC) compounds for fuel cells.^1−5^ Transition metal catalysts, e.g., based on platinum and rhodium, have demonstrated to improve the reactivity for these reactions.^6−9^ However, several challenges such as coke formation and limited selectivity for the production of specific alkenes due to consecutive dehydrogenation are reported.^10,11^ Theoretical models, specifically the d-band theory developed by No̷rskov and co-workers,^12−14^ somewhat reasonably describe for these reactions the correlation between electronic structure (d-band center), binding strength of the catalysts and adsorbates (C, H, O, etc.), and activation energy for breaking C–H bonds. This provides a foundation on how to discuss the reaction mechanisms systematically and screen catalytically active species aiming at improving productivity and selectivity for these dehydrogenation reactions.^12,15−17^ In this framework of understanding the underlying alkane dehydrogenation mechanisms, the low reactivity can be attributed to the weak binding strength between catalytically active sites and the adsorbed carbon atoms, decreasing the possibility of triggering subsequent chain reactions, and undesired side reactions (e.g., alkyne and coke formation) causing low selectivity are rationalized by hampered desorption of alkene products.^1,16,18,19^ According to previous studies about the d-band and its impact on catalytic reactions, tailoring the d-band of active sites may indeed play an important role in insight-driven catalyst development.

GaRh-based supported catalytically active liquid metal solutions (SCALMS) have been demonstrated to be efficient catalysts for propane dehydrogenation reactions, i.e., reaching up to 30% of conversion and 95% selectivity at 550 °C.^20−23^ GaRh SCALMS, consisting of low concentrations (<1%) of Rh atoms dispersed in a *liquid* Ga matrix, show an extraordinary reactivity and coking resistance in propane dehydrogenation reactions compared to conventional Rh/Al_2_O_3_ catalysts.^20,24^ It has been suggested that the Rh atoms at relevant reaction conditions are dispersed in a *liquid* matrix of Ga and thus act as isolated catalytically active sites, being capable of stabilizing certain intermediates that promote alkene production and suppress coking.^18^

Studies of the electronic structure of solid GaRh model systems show Rh 4d-derived states in the valence band. The corresponding spectral fingerprint narrows and shifts to higher binding energies (BE, i.e., moving away from the Fermi level, *E*_F_) with a decreasing amount of Rh in the Ga matrix.^20,25,26^ Based on accompanying DFT calculations, this spectral change has been suggested to be an indication for the site isolation of Rh atoms.^25,27^ According to the work from No̷rskov et al., the activation energy for catalytic reactions is highly correlated with the binding strength between catalysts and reactants, which is affected by the electronic structure of the catalytically active sites.^12,14,17^ Varying the character (position and width) of the transition metal derived d-states will change the occupancy of the hybrid antibonding state and alter the catalyst-adsorbate binding strength.^1,12,28,29^ According to these studies, isolated Rh 4d-derived sites showing a narrow d-band feature around 3 eV are hence supposed to bound weaker to reactants, which can be an origin for the observed enhanced reactivity.^25,27^

However, the published results on the electronic structure of GaRh model systems might have limited relevance for the true properties of the catalytically active surface of real-world GaRh SCALMS under reaction conditions. In particular, the chemical and electronic structures of Rh atoms in a liquefied Ga matrix, which is highly relevant for the catalytic reactivity and selectivity, have not yet been reported. GaRh SCALMS starts to show increased reactivity at around 550 °C, a temperature at which the Ga matrix is liquid. In addition to the high affinity of Ga toward oxygen,^30,31^ this might further promote surface oxidation.^32^ Microscopy studies indicate the growth of a GaO_*x*_ shell upon exposure of GaRh alloy nanoparticles to ambient conditions.^26^ However, GaO_*x*_ can partially be removed by annealing in ultrahigh vacuum (UHV)^20,26,33−35^ at temperatures at which the Ga matrix is expected to be liquid.^36^ Thus, a complex interplay can be expected, requiring a detailed study of the influence of surface oxidation and liquefaction on the electronic structure of GaRh alloys.

In this study, the effects of surface oxidation and GaO_*x*_ removal treatments (at liquefying temperatures) on the chemical and electronic structure of SCALMS catalysts is addressed by means of examining a model system consisting of 7 at. % Rh containing GaRh alloy nanoparticles on a SiO_*x*_/Si support by *in-system* X-ray photoelectron spectroscopy (XPS) and ultraviolet photoelectron spectroscopy (UPS) measurements (i.e., sample preparation and characterization is done in one interconnected multichamber UHV system, avoiding air exposure between synthesis and measurement). Our spectroscopic analysis indeed suggests that isolated Rh atoms are present at relevant reaction conditions while accompanying scanning electron microscopy (SEM) measurements reveal that at the same time, the GaRh sample undergoes drastic structural changes, insights that are crucial for a further deliberate optimization of SCALMS catalysts.

## Material and Methods

Fully exploiting the synthesis and characterization capacities of the Energy Materials In-Situ Laboratory Berlin (EMIL) that is composed of an interconnected system of different UHV chambers,^37^ sample preparation as well as the oxidation/annealing treatments, and spectroscopic measurements have been performed “in-system”, i.e., without exposing the sample to ambient conditions to avoid undesired surface oxidation and contamination of the studied GaRh samples by air and/or moisture.

### Sample Preparation

Gallium (powder, 99.99%) and rhodium (wire, 99.95%) were purchased from Sigma-Aldrich/Alfa Aesar, respectively. The nanostructured GaRh alloy samples were prepared via physical vapor deposition (PVD) using a SPECS EBE-4 e-beam evaporator. All GaRh samples were prepared in UHV at a base pressure <1 × 10^–8^ mbar by coevaporation of Ga and Rh on a silicon wafer (boron-doped, Czochralski tech. prepared, 2–4 Ω·cm resistance) with native silicon oxide (SiO_*x*_/Si). Substrates were annealed to 500 °C to clean the surface and desorb water before deposition. For studying potentially present substrate-induced oxidation, the native silicon oxide was removed from the Si wafer by Ar^+^-ion sputtering using energy of 1 kV and filament current of 10 mA at 1 × 10^–5^ mbar Ar condition for 30 min before GaRh deposition. During deposition, the substrate was kept at room temperature. Rh was evaporated using a 1 mm-thick wire, and Ga was evaporated from a BN crucible. The deposition rate was monitored using a quartz crystal microbalance. GaRh alloys with Rh contents of 7 at. % with a nominal film thickness of approximately 30 nm have been prepared. For photoemission measurements, samples were transferred under UHV conditions in between preparation and analysis chamber after deposition and oxidation/annealing treatments.

### Sample Oxidation and GaO_*x*_ Removal

The surface oxidation experiments were conducted in the same chamber as the sample preparation by means of stepwise exposure of the GaRh sample to 1 × 10^–6^ mbar of oxygen, resulting in accumulated oxidation times of 10, 30, 60, and 240 min while keeping the sample at room temperature. After each step, the sample was transferred under UHV conditions to the analysis chamber (base pressure ≈ 2 × 10^–9^ mbar) for the photoemission measurements. After the measurements of the GaRh sample oxidized for a total of 240 min, the (GaO_*x*_ removing) annealing experiments are conducted directly in the analysis chamber (base pressure ≤5 × 10^–9^ mbar) at temperatures of 300, 400, 600, and 650 °C while performing in-situ photoemission measurements. Annealing beyond 650 °C is not conducted due to the risk of contamination of the surface analysis system by evaporation of significant amounts of Ga.

### Photoemission Spectroscopy

X-ray (XPS) and ultraviolet (UPS) photoelectron spectroscopy measurements were conducted using nonmonochromatized Mg *K*_α_ (1253.56 eV) irradiation from a SPECS XR 50 X-ray source and He II (40.8 eV) irradiation from a Prevac UVS 40A2 gas discharge lamp, respectively. The photoelectrons were detected by a Scienta Omicron Argus CU electron analyzer. The pass energy for the core-level detailed spectral measurements was set to 20 eV, resulting in a total experimental energy resolution of approximately 1.0 ± 0.3 eV for Mg *K*_α_ XPS. For the He II-UPS measurements, a pass energy of 5 eV was used, resulting in a total experimental resolution of approximately 0.2 ± 0.05 eV (see the Supporting Information, for more details). For the He II-UPS measurements conducted at 650 °C, we do observe a thermal broadening, resulting in a total experimental resolution of approximately 0.22 eV in this case. The BE of the XPS and UPS measurements was calibrated by the Fermi edge (*E*_F_) of a clean Au foil. The details of XPS/UPS data analysis are elaborated in the Supporting Information (SI).

### SEM

Selected samples were examined by SEM measurements using a Hitachi S-4100. For this, the sample to be studied was transferred under ambient conditions from the sample preparation chamber to the SEM setup. The SEM images are processed via ImageJ for the determination of particle size distributions. Two hundred particles in the center region of the SEM image were selected for the size distribution analysis.

## Results and Discussions

### Evolution of the Chemical Structure upon Oxidation

**Figure 1:**
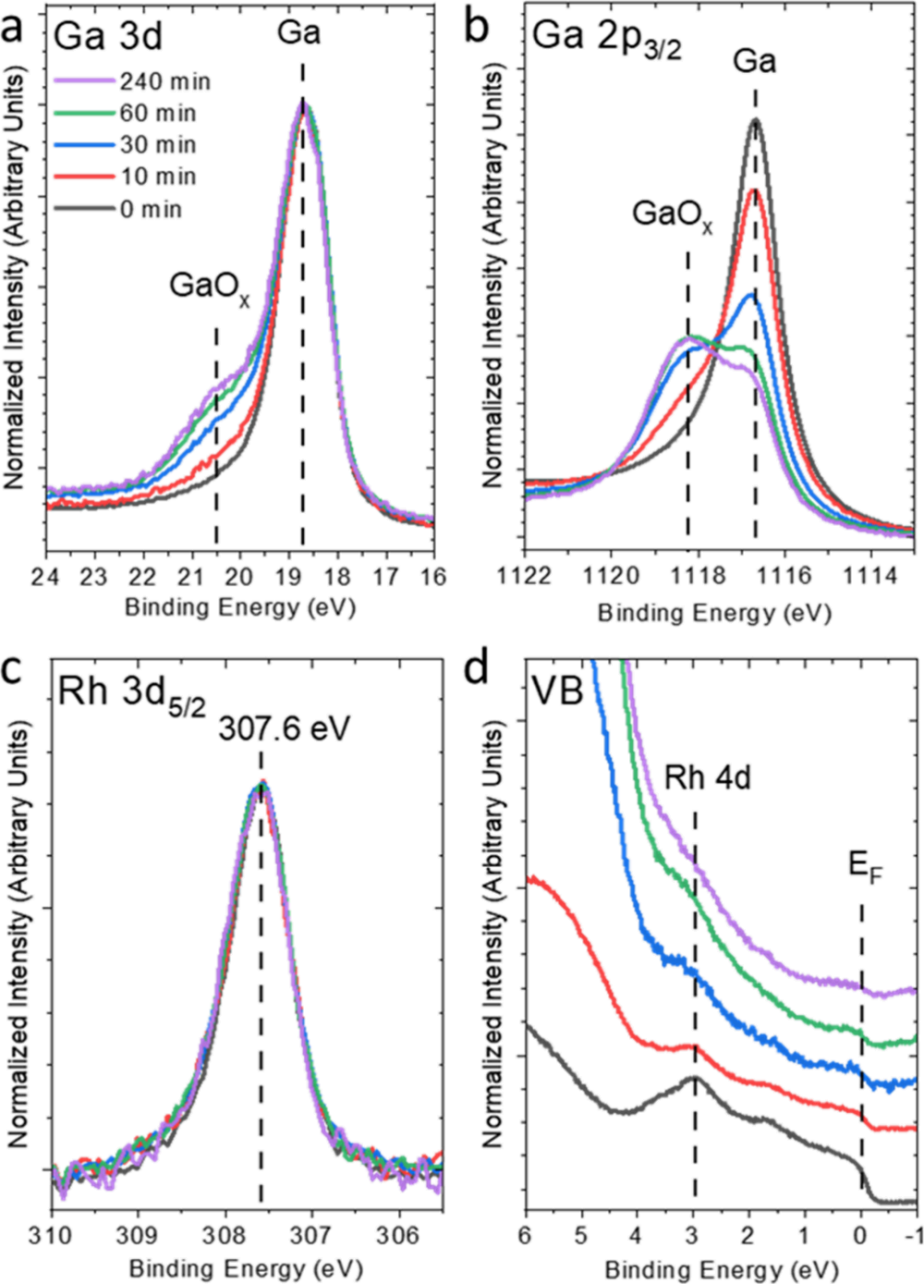
Mg *K*_α_XPS spectra of the (a) Ga 3d, (b) Ga 2p_3/2_, and (c) Rh 3d_5/2_ core levels of the GaRh alloy sample containing 7 at. % Rh in the as-prepared state (referred to as “0 min” exposure) and after 10, 30, 60, and 240 min exposure to 1 × 10^–6^ mbar O_2_. (d) The corresponding He II-UPS spectra of the valence band (VB), stacked for clarity. The vertical dashed lines indicate different chemical species, peak positions, or prominent spectral features (e.g., the Fermi edge [*E*_F_] in (d)). Corresponding extended UPS spectra up to 10 eV BE are shown in Figure S1.

The Ga 3d and 2p_3/2_ as well as Rh 3d_5/2_ core level and valence band (VB) XPS spectra are shown in Figure 1. For the as-prepared GaRh sample with 7 at. % Rh (referred to as “0 min” oxidation in Figure 1a,b), the Ga 3d and Ga 2p_3/2_ peaks are dominated by single spectral contributions at BEs of 18.6 and 1116.7 eV, respectively, which we attribute to metallic Ga. Note that the corresponding XPS survey spectrum is dominated by Ga- and Rh-related photoemission and Auger lines (see the SI, Figure S2a) confirming a contamination-free sample surface (most prominently demonstrated by the missing C 1s line at 285 eV; see Figure S2c). This indicates the success of the in-system approach of performing sample preparation and characterization in one UHV system, avoiding exposure to ambient conditions between sample synthesis and analysis. However, minor spectral features assigned to oxygen (O 1s and O KLL, see the SI, Figure S2a) can be observed. The main oxygen contribution comes from the SiO_*x*_/Si support that is not completely covered by the GaRh nanoparticles (Figures S2b and S3a). Furthermore, close inspection of the data reveals minor additional contributions at higher BE for the Ga-related core levels, in particular for the Ga 2p_3/2_ peak at 1118.2 eV (see the Supporting Information, Figure S6 for detailed peak fitting results), which we ascribe to GaO_*x*_. In contrast, the Rh 3d_5/2_ spectrum can be described by a single contribution at 307.6 eV in line with previous results studying isolated Rh sites in GaRh alloys,^25,27^ without any indication for the presence of an oxidized Rh species. The spectral change only observed in the Ga 2p_3/2_ core level corroborates the higher affinity of Ga toward oxygen than Rh.^30,31^ The oxygen in our case presumably comes from the residual oxygen gas in the UHV deposition/analysis chambers or the SiO_*x*_/Si support (see discussion below).

Upon exposing the sample to 1 × 10^–6^ mbar partial pressure of oxygen, new Ga 3d and Ga 2p_3/2_ features at 20.5 and 1118.2 eV BE, respectively, attributed to Ga–O bond formation (Figure 1a,b) appear and increase in intensity with exposure time. At the same time, we find a minor increase of the C 1s line intensity (see the SI, Figure S2c). Together with the corresponding XPS survey spectra that exclusively show Ga-, Rh-, and O-related photoemission and Auger features (see the SI, Figure S2a), this indicates that the high quality of the sample (surface) is maintained also upon performing the oxidation treatments. Comparison of the Ga 3d and Ga 2p_3/2_ spectra reveals that the increase of the spectral feature related to Ga–O bonds is more pronounced for the latter (see also the detailed fit analysis in the SI, Figures S5 and S6). This is due to the different inelastic mean free path (IMFP) of the Ga 3d (IMFP = 20.9 Å) and Ga 2p_3/2_ (IMFP = 5.3 Å) photoelectrons and indicates that the oxidation mainly takes place at the GaRh surface.^38−41^ The details of XPS peak fitting with reported IMFP and photoionization cross-section (σ) are elaborated in the experimental section in the SI. Note that the fit of the Ga–O feature shown in the SI encompasses all present gallium oxide (GaO_*x*_) species. We use a peak that is significantly broader than that ascribed to metallic Ga, justifying this by acknowledging that this peak may contain different Ga–O species (which we summarize and refer to as GaO_*x*_ in the following), such as substoichiometric Ga_2_O_3−δ_ (as also discussed in ref (42)) and stoichiometric Ga_2_O_3_, rather than a single oxide state at a specific BE. In any case, there are only minor changes in the Ga 3d and Ga 2p_3/2_ spectra between 60 and 240 min oxygen exposure, which we presumably interpret as a self-limited GaO_*x*_ formation, as also observed for pure Ga and GaPt samples.^42,43^ Assuming a simple GaO_*x*_/Ga bilayer model with a closed GaO_*x*_ film that homogeneously covers the Ga, the oxide film thickness after 240 min exposure to 1 × 10^–6^ mbar partial pressure of oxygen is estimated to be (7.3 ± 1.0) and (9.0 ± 1.0) Å calculated from Ga 2p_3/2_ and Ga 3d, respectively (see also SI 1.3 for details), which corresponds to the thickness between one and two monolayers of GaO_*x*_ (see also quantified results of the Ga 3d and Ga 2p_3/2_ fits in the SI, Tables S1 and S2).^42^ The formation of ultrathin GaO_*x*_ layers was observed previously for Ga, GaPd, and GaPt oxidized in 10^–5^–10^–7^ mbar partial pressures of oxygen and was explained by limited mass transport of oxygen into the subsurface of Ga.^26,42,44^

The Rh 3d_5/2_ spectra seem to be unaffected by the oxidation treatment and can still be described by one spectral contribution remaining at 307.6 eV (see Figure 1c), mainly indicating a preservation of the chemical structure of the Rh within the GaRh alloy despite exposure to 1 × 10^–6^ mbar O_2_. We assume that, as in our previous study,^27^ the feature at 307.6 eV in the spectrum of the 7 at. % Rh sample at room temperature represents multiple GaRh species including high melting point GaRh intermetallic compounds (IMCs, presumably Ga_21_Rh_4_). For extended oxidation times (30, 60, and 240 min), there is some indication for the presence of a small second component at higher BE of 308.5 eV, which is 1.3 eV higher than pure Rh film (307.2 eV),^27^ which can be seen from the fitting results (see Figure S7). In a previous study, this second Rh species was attributed to Rh located within the GaO_*x*_ layer,^26^ presumably indicating the formation of Rh–O bonds. Accordingly, a 1.2–1.3 eV shift of the Rh 3d peak to higher BE caused by Rh oxidation is reported.^45^

The Rh concentration (derived based on the Rh 3d_5/2_ and Ga 3d XPS data, see Table S3) remains at 7.0 ± 0.5 at. % after the first few oxidation steps (0, 10, and 30 min), indicating the preservation of the amount of Rh in the probed volume. In subsequent oxidation steps (60 and 240 min), the Rh concentration decreases to 5.3 ± 0.5 at. % (Table S3). Such Rh depletion suggests that at these oxidation conditions, most Rh atoms are buried by the GaO_*x*_ shell, in agreement with finding only a small amount of the second (O–Rh bond related) Rh 3d_5/2_ component in corresponding spectra (see Figure S7). Note that the Rh concentrations obtained based on the Rh 3d_5/2_ and Ga 3d (see Table S3) and based on the Rh 3d_5/2_ and Ga 2p_3/2_ (see Table S4) XPS data are significantly different. The Rh concentration derived using the surface-sensitive Ga 2p_3/2_ line is derived to be 2.5 ± 1 at. %, which we attribute to a significant (Rh/Ga) depth profile in the surface region of the GaRh samples. This is corroborated by DFT-based molecular dynamics simulations that suggest the very surface of a GaRh slab to be Rh-free. The observed Rh concentration decreasing contradicts previous findings about Rh enrichment due to surface oxidation of macroscopic GaRh samples.^26^ However, the morphology of macroscopic GaRh samples and spatial distribution of Rh atoms is different from our codeposited nanostructured GaRh sample; hence, finding a different Rh concentration profile is reasonable.

Previously, we also identified the Rh 4d-derived states in the VB of GaRh samples to be sensitive markers for the presence of isolated Rh atoms.^27^ In fact, the UPS spectrum of the as-prepared (“0 min”) GaRh sample in Figure 1d resembles quite well that of the corresponding sample in our previous publication.^27^ Thus, we attribute the distinct (narrow) spectral feature related to Rh 4d-derived states at around 3 and 1.5 eV in the UPS VB spectrum to isolated Rh sites. With increasing oxidation time, the *E*_F_-related feature (at BE = 0 eV) indicative for the metallic nature of Ga and the Rh 4d-related feature decrease in intensity while the spectral feature at around 6 eV BE related to O 2p-derived states, caused by the growing GaO_*x*_ shell, increases (see Figure 1d).^46^ Up to an exposure time of 30 min, the Rh 4d-related spectral feature can still be clearly observed in the corresponding UPS VB spectra. Because of the O 2p-related spectral intensity that becomes dominating for the GaRh sample exposed to 1 × 10^–6^ mbar O_2_ for 60 and 240 min, it is not so clear whether the spectral feature attributed to Rh 4d-derived states around 3 eV BE is still visible in these cases. However, the direct comparison with data from ref (42) of a similarly oxidized Ga sample without Rh in Figure S8 suggests that even for these extended oxidation times, there is some Rh 4d-related spectral intensity around 3 eV BE, indicating the preservation of isolated Rh sites at the sample surface even after prolonged oxidation.

### Evolution of the Chemical and Electronic Structure upon Annealing-Induced GaO_*x*_ Removal

**Figure 2:**
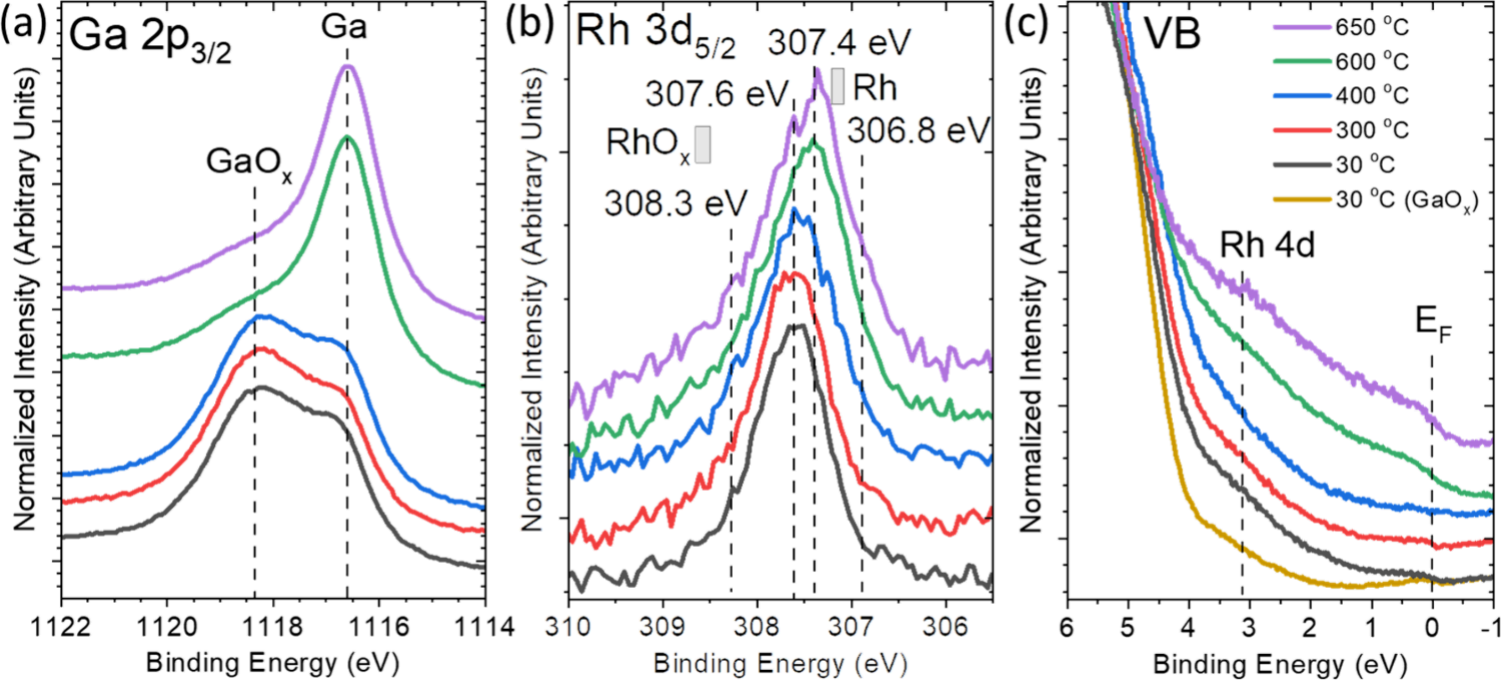
Mg *K*_α_-excited (a) Ga 2p_3/2_, (b) Rh 3d_5/2_ XPS, and He II-excited (c) UPS spectra of the valence band (VB) of a GaRh alloy sample containing 7 at. % Rh oxidized for 240 min annealed to (and measured at) different temperatures. Reference Rh 3d_5/2_ BE positions of Rh (as reported in ref (27)) and of RhO_*x*_ (+1.3 eV compared to Rh, as suggested in ref (45)) are indicated by gray rectangles in Figure 2b. The VB spectrum of oxidized pure Ga (“GaO_*x*_”, from ref (42)) is shown in the orange line as reference in Figure 2c. The vertical dashed lines indicate different chemical species, peak positions, or prominent spectral features (e.g., the Fermi edge [*E*_F_] in (c)).

According to previous studies, the gallium oxide layer formed during synthesis of GaRh alloys can be removed by annealing at a high temperature (≈600 °C) in a vacuum environment (10^–7^ mbar).^20,26^ Note that at this temperature, the Ga matrix is liquid.^47^ The removal of the surface oxide layer is considered as an activation step for real-world GaRh SCALMS. Microscopy results suggest that the oxide layer removal can be explained by the formation and evaporation of volatile Ga_2_O.^20,26,33^ However, the chemical and electronic structure of the isolated Rh atoms at corresponding liquid GaRh surfaces after gallium oxide removal has remained an open question. Thus, XPS and UPS measurements have been performed during annealing at temperatures between 30 and 650 °C to mimic the activation step of real-world GaRh SCALMS and to reveal the properties of the activated (liquid) GaRh surface (Figure 2). Morphological changes of the GaRh alloy samples upon annealing are observed via SEM (see corresponding images recorded ex-situ after cooling down in Figure S3). After annealing to 650 °C, the topography of the GaRh sample on SiO_*x*_/Si changes from being best described as a mixture of nanoparticles with bimodal size distribution, i.e., similar to the topography of Ga/SiO_*x*_/Si samples,^42^ and agglomerated (bigger) particles with a polygonal shape (Figures S3 and S4).

The surface oxide removal is documented by the decreasing GaO_*x*_ feature of the Ga 2p_3/2_ line once the (oxidized) GaRh alloy is annealed to 600 °C (Figure 2a), i.e., a temperature at which the GaRh alloy is expected to be liquid.^48^ The calculated GaO_*x*_ thickness reduces from 7.3 ± 1.0 to 2.6 ± 0.5 Å when the temperature reaches 600 °C and further decreases to 2.0 ± 0.5 Å when temperature ramps up to 650 °C, indicating an efficient removal, i.e., of 73% of the GaO_*x*_ surface layer (Table S5). The mechanism for GaO_*x*_ removal is reported as taking place via the formation of Ga_2_O, which is volatile even in ambient conditions.^36,49^ However, even for an annealing temperature of 650 °C, we can still clearly identify spectral intensity at the high BE side of the Ga 2p_3/2_ line attributed to GaO_*x*_ (see Figure 2a and Figure S9). The residual GaO_*x*_ is also observed in the GaCu system.^34,35^ Note that for the GaRh samples annealed at 600 and 650 °C, the detailed fit analysis reveals an additional Ga 2p_3/2_ spectral contribution at 1118.7 eV, which we attribute to a different Ga–O species (“Ga–O_2”; Figure S9), which is also observed when annealing Ga in UHV on a SiO_*x*_/Si support^42,50^ and in agreement with earlier reports on the formation of gallium oxide species as a result of the reaction of gallium with quartz at elevated temperatures.^49^ This suggests that (also) the substrate can act as an oxygen source, enabling substrate-induced oxidation, a topic that is discussed in detail below.

**Figure 3:**
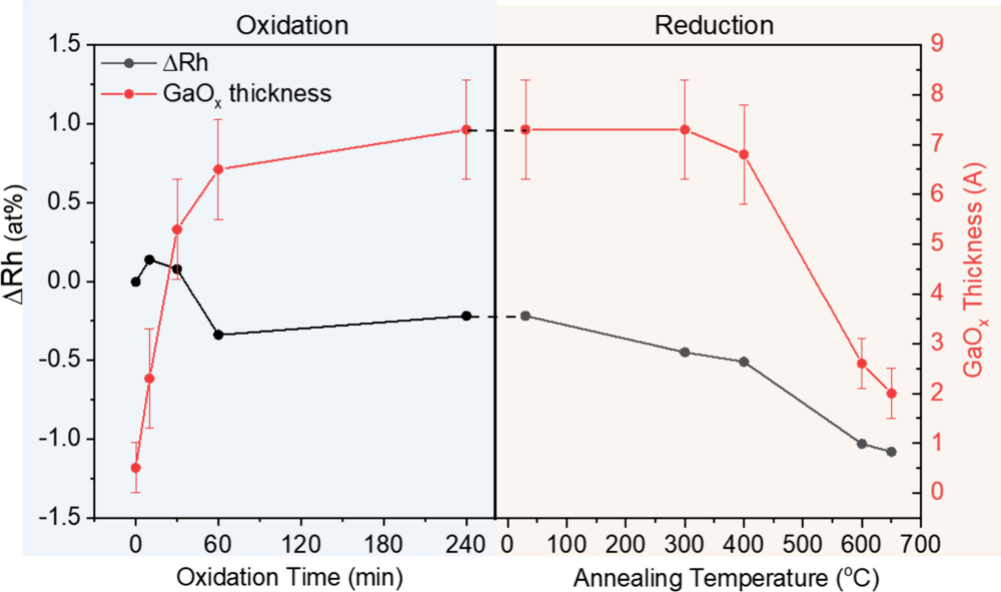
Evolution of the calculated GaO_*x*_ thickness in Å (red) and change of the Rh concentration (black) relative to the as-prepared GaRh alloy sample nominally containing 7 at. % Rh (ΔRh) upon surface oxidation (in 1 × 10^–6^ mbar O_2_, left panel) and annealing-induced reduction (in vacuum conditions, right panel) treatments. Quantification is based on fitting results of Rh 3d_5/2_ and Ga 2p_3/2_ core-level spectra shown in Figures S6, S7 and Figures S9, S10.

Upon annealing to 400 °C, the Rh 3d_5/2_ spectra (Figure S10a,b) are very similar to that recorded for the oxidized GaRh sample before annealing (Figure S7e). Significant changes in the Rh 3d_5/2_ core level peak including the main peak shift from 307.6 to 307.4 eV and the appearance and increase of two new spectral features are observed for temperatures of 600 °C and above (Figure 2b and Figure S10). The C 1s/Ga LMM spectra are very similar for the as-prepared sample and the same sample measured at 650 °C (Figure S11), ruling out the carbon contamination as an explanation for the observed spectral changes in the Ga 2p_3/2_ and Rh 3d_5/2_ spectra. We find the two new Rh 3d_5/2_ components at 308.3 eV (“Ga–Rh_2”) and 306.8 eV (“Ga–Rh_3″, see Figure S10c,d). The peak ascribed to the O–Rh bonds (308.5 eV) cannot properly represent the spectral feature at the high BE region of the Rh 3d_5/2_ peak measured at 600 °C and above. This is in line with the lower reduction energy barrier of RhO_*x*_ compared to GaO_*x*_ corroborated by RhO_*x*_ reduction experiments in CO environments, suggesting that the promotion of RhO_*x*_ formation during GaO_*x*_ reduction is unlikely.^51−54^ Detailed analysis of different fit approaches results in the addition of the “Ga–Rh_2” feature with different peak positions and shapes (asymmetric Doniach-Sunjic rather than Voigt profile) in the fit, resulting in the Rh–O contribution to become negligible. In a previous study,^27^ we deliberately varied the Rh content and, in the range of 55 to 3 at. % Rh, observed a corresponding Rh 3d_5/2_ shift from 306.9 to 307.6 eV; therefore, we attribute the “Ga–Rh_2” Rh 3d_5/2_ component to represent a Rh-depleted GaRh alloy and the “Ga–Rh_3” Rh 3d_5/2_ component to be indicative for a Rh-rich GaRh alloy.^20,27^ The BE of the new Rh 3d_5/2_ contribution “Ga–Rh_2” is 0.7 eV higher than the Rh 3d_5/2_ peak of the 3 at. % GaRh sample in ref (27), and thus, we speculate that this contribution represents a part of the studied GaRh sample that has a much lower content of (isolated) Rh sites. The “Ga–Rh_3” feature becomes more pronounced upon increasing the temperature to 650 °C, indicating an enhanced formation of Rh-rich GaRh alloy at this temperature (Figure 2b and Figure S10d). The evolution of GaO_*x*_ thickness and the change in Rh concentration (ΔRh) during surface oxidation and annealing are summarized in Figure 3; the detailed fitting results that are the base for this quantification are shown in Figures S6, S7, S9, and S10 and summarized in Tables S4–S6. After 60 min of oxidation, a one-to-two-monolayer-thick GaO_*x*_ shell is formed, burying most of the Rh. During annealing, the GaO_*x*_ shell is efficiently removed when the annealing temperature reaches 600 °C.

A close inspection of the VB spectra in Figure 2c reveals an overall increase of the (background) intensity between 4 and 0 eV BE upon annealing at 600 °C and above, i.e., the temperature where efficient removal of the GaO_*x*_ is observed. This becomes particularly apparent when comparing the data with the VB spectrum of a (Rh-free) GaO_*x*_ sample.^42^ For the high-temperature measurements of the GaRh sample, the Fermi edge (*E*_F_) reemerges, indicating the reappearance of the metallic nature of the Ga matrix at the surface. A broadening of the Fermi-edge width by 0.06 eV is expected for the measurement at 650 °C compared to that at room temperature. Nevertheless, an extra 0.29 eV Gaussian broadening of the spectrum of the 7 at. % Rh-containing nanostructured GaRh alloy sample oxidized in 1 × 10^–6^ mbar O_2_ for 10 min (from Figure 1d) is required to achieve a reasonable agreement with the Fermi-edge width of the 650 °C measurement (see Figure S12b), which we currently attribute to either He II excitation satellite-related spectral intensity around *E*_F_ or the formation of multiple GaRh species during annealing, resulting in the superposition of different spectral features broadening the spectrum. The reemergence of the Rh 4d-derived feature is less pronounced, as clearly shown by the comparison of the VB spectra of the GaRh sample measured at 650 °C and the sample that had been oxidized for 10 min (Figure S12a), i.e., samples that should have a similar GaO_*x*_ content (as shown in Figure 3). The reason for the suppressed appearance of the Rh 4d feature can be attributed to the thermal broadening as elaborated above (and discussed in detail in conjunction with Figure S12b), to a lower Rh content (see Figure 3) and to peak broadening due to the formation of multiple GaRh species (as evident from the multiple contributions to the Rh 3d_5/2_ spectrum in Figure 2b). We find a good agreement between the (deliberately additionally) broadened spectrum of the 10 min oxidized GaRh sample and the spectrum measured at 650 °C not only with respect to the Fermi edge but also for the Rh 4d-derived spectral region (see details in Figure S12b), supporting the conclusion that isolated Rh atoms reemerge at the sample surface at these temperatures. Note that the applied Gaussian broadening of 0.35 eV can only to a minor extent be attributed to thermal broadening (only 0.06 eV thermal broadening is expected when increasing the measurement temperature from room temperature to 650 °C); the majority of the broadening is tentatively ascribed to the presence of multiple species having spectral intensity around the Fermi edge. The remaining high (background) intensity at 6 eV for the 600 and 650 °C measurements is caused by O 2p-derived states from residual (substrate-induced) GaO_*x*_; see discussion below.

### Substrate-Induced Ga Oxidation

**Figure 4:**
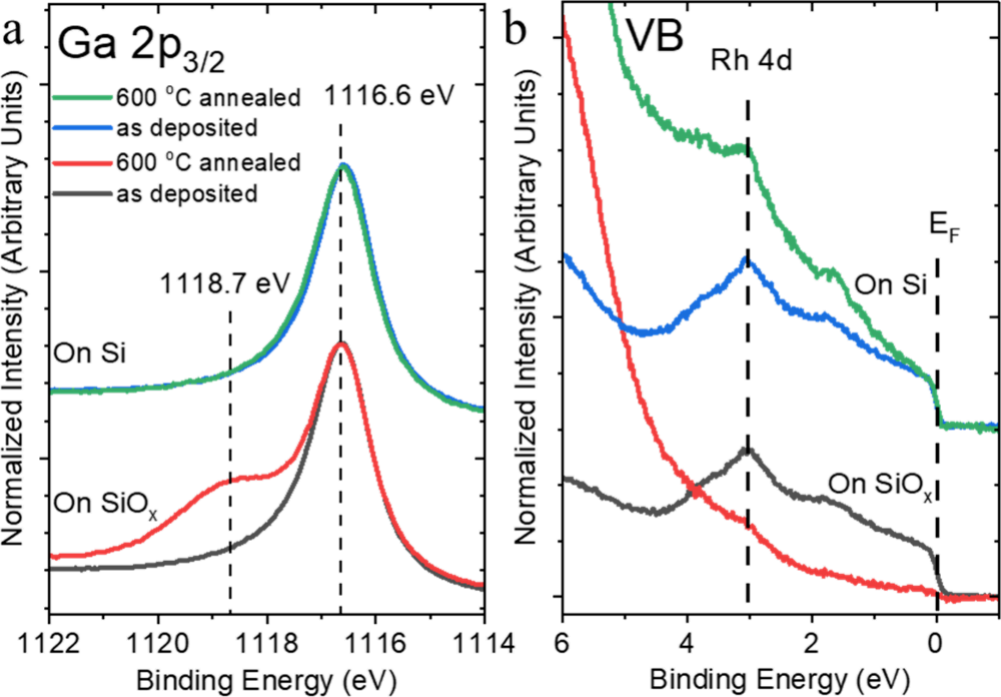
(a) Mg *K*_α_ XPS Ga 2p_3/2_ and (b) He II-UPS VB spectra of a GaRh alloy sample nominally containing 7 at. % Rh PVD deposited on sputter-cleaned Si and SiO_*x*_/Si substrates before (as-prepared) and after annealing at 600 °C at a base pressure ≤10^–8^ mbar condition for 30 min. GaRh on SiO_*x*_/Si: black and red lines; GaRh on sputter-cleaned Si: blue and green lines.

Understanding the origin of residual GaO_*x*_ (present even at temperatures >600 °C) is critical for catalyst development. As it is reported that metallic Ga can reduce SiO_*x*_ and form GaO_*x*_ at high temperatures,^55^ also catalyst–substrate interaction needs to be considered. For this purpose, a bare Si substrate without a native oxide layer was prepared; the SiO_*x*_ was removed by Ar^+^-ion sputtering. The XPS survey spectrum of the sputter-cleaned Si wafer compared to that of a natively oxidized Si wafer indeed shows a greatly reduced O 1s line (see Figure S13a). However, with similar C 1s line intensities and after Ar^+^-ion sputtering, additional Ar- and N-related peaks can be identified in the survey spectra, indicating that the sputtered Si sample is far from having an ideal surface. In any case, the Si 2p XPS spectrum of the sputter-cleaned Si wafer mainly exhibits one peak at 99.5 eV, indeed indicating (an almost complete) SiO_*x*_ removal (Figure S13b). For a direct comparison, GaRh containing 7 at. % Rh was deposited on the sputter-cleaned Si and on the SiO_*x*_/Si substrates without breaking vacuum conditions between substrate cleaning and GaRh alloy deposition. The comparison of the corresponding XPS and UPS data before and after annealing at 600 °C (measured after cooling down to room temperature) is shown in Figure 4. The Ga 2p_3/2_ core-level peak of GaRh alloy deposited on the SiO_*x*_/Si substrate shows the formation of Ga–O bonds upon annealing, which contrasts with the 7 at. % Rh containing nanostructured GaRh alloy deposited on sputter-cleaned Si. Interestingly, the spectroscopic Ga 2p_3/2_ feature of the formed Ga–O bonds is different from monolayered GaO_*x*_ grown upon deliberate oxidation in 1 × 10^–6^ mbar O_2_ shown in and discussed in conjunction with Figure 1. The BE position of this Ga–O feature is at 1118.7 eV (see “Ga–O_2” in Figure S14), which is 0.6 eV higher than the Ga–O feature observed for the deliberate surface oxidation experiments in partial pressure of O_2_ (1118.2 eV, see Figure 1a and Figure S6 and termed “Ga–O_1” in Figure S14). The “Ga–O_2” feature is also observed in the Ga 2p_3/2_ spectrum of the 650 °C annealing sample and becomes more pronounced, suggesting that this Ga–O bonding comes from a GaO_*x*_ species formed by substrate-induced gallium oxidation (Figure S9d). The topography of the GaRh alloy on the different substrates after annealing is quite different (see Figure S15). This can be explained by the GaRh alloy’s ability to wet the hydrophobic Si surface much better than the hydrophilic SiO_*x*_ surface. In conclusion, our data confirms that SiO_*x*_ can act as an oxygen source resulting in a Ga oxidation during annealing at 600 °C in vacuum, explaining the inability to completely remove GaO_*x*_ from the GaRh/SiO_*x*_/Si system even at vacuum annealing temperatures >600 °C, as discussed in conjunction with Figure 2.

## Conclusions

A comprehensive X-ray/UV photoelectron spectroscopy and SEM study of a 7 at. % Rh containing nanostructured GaRh alloy on a SiO_*x*_/Si support as a model system for real-world SCALMS has been conducted to reveal the chemical and electronic structures and sample morphology changes upon performing oxidation and annealing treatments mimicking relevant reaction conditions. A decrease in the total Rh surface concentration during oxidation suggests that Rh atoms are buried below the growing GaO_*x*_ shell. A successful GaO_*x*_ removal is observed upon annealing at 600 °C under vacuum conditions. At high temperatures, two new Rh species can be identified, which we attribute to Rh-depleted and Rh-rich GaRh species. Together with the reemergence of Fermi edge and Rh 4d-derived spectral features in the VB spectra upon annealing, we interpret this as being indicative for the presence of metallic Ga and the formation of a GaRh species with isolated Rh atoms. Furthermore, the presence of a residual GaO_*x*_ species is always observed even when the sample is annealed at 650 °C, which is attributed to substrate-induced oxidation. These insights will help to pave the way for further insight-driven optimization of SCALMS systems.

## Data Availability

The XPS and SEM data in this study are openly available in Zenodo at: DOI/10.5281/zenodo.10580028.

## Supporting Information Available

Description of XPS peak analysis procedure; SEM images and analysis; additional XPS/UPS spectra of GaRh alloys after oxidation and annealing; Ga 2p_3/2_, Ga 3d, and Rh 3d_5/2_ XPS fitting results; and quantified XPS fitting results in value (PDF)

## References

[ref1] Chang X.; Lu Z.; Wang X.; Zhao Z.-J.; Gong J. Tracking C–H Bond Activation for Propane Dehydrogenation over Transition Metal Catalysts: Work Function Shines. Chem. Sci. 2023, 14, 6414–6419. 10.1039/D3SC01057K. 37325145 PMC10266452

[ref2] Solymosi T.; Auer F.; Dürr S.; Preuster P.; Wasserscheid P. Catalytically Activated Stainless Steel Plates for The Dehydrogenation of Perhydro Dibenzyltoluene. Int. J. Hydrogen Energy 2021, 46, 34797–34806. 10.1016/j.ijhydene.2021.08.040.

[ref3] Modisha P.; Gqogqa P.; Garidzirai R.; Ouma C. N. M.; Bessarabov D. Evaluation of catalyst activity for release of hydrogen from liquid organic hydrogen carriers. Int. J. Hydrogen Energy 2019, 44, 21926–21935. 10.1016/j.ijhydene.2019.06.212.

[ref4] Müller K.; Skeledzic T.; Wasserscheid P. Strategies for Low-Temperature Liquid Organic Hydrogen Carrier Dehydrogenation. Energy Fuels 2021, 35, 10929–10936. 10.1021/acs.energyfuels.1c01170.

[ref5] Sattler J. J. H. B.; Ruiz-Martinez J.; Santillan-Jimenez E.; Weckhuysen B. M. Catalytic Dehydrogenation of Light Alkanes on Metals and Metal Oxides. Chem. Rev. 2014, 114, 10613–10653. 10.1021/cr5002436. 25163050

[ref6] Adlhart C.; Uggerud E. Mechanisms of Catalytic Dehydrogenation of Alkanes by Rhodium Clusters Rhn+ Probed by Isotope Labelling. Int. J. Mass spectrom. 2006, 249–250, 191–198. 10.1016/j.ijms.2005.12.032.

[ref7] Chen X.; Peng M.; Cai X.; Chen Y.; Jia Z.; Deng Y.; Mei B.; Jiang Z.; Xiao D.; Wen X.; Wang N.; Liu H.; Ma D. Regulating Coordination Number in Atomically Dispersed Pt Species on Defect-rich Graphene for n-Butane Dehydrogenation Reaction. Nat. Commun. 2021, 12, 2664 10.1038/s41467-021-22948-w. 33976155 PMC8113322

[ref8] Biloen P.; Dautzenberg F. M.; Sachtler W. M. H. Catalytic Dehydrogenation of Propane to Propene over Platinum and Platinum-Gold Alloys. J. Catal. 1977, 50, 77–86. 10.1016/0021-9517(77)90010-0.

[ref9] Shih K. C.; Goldman A. S. Alkane Dehydrogenation Catalyzed by Rhodium(I) Phosphine Complexes: Observation of The Stoichiometric Alkane-to-Rhodium Hydrogen-Transfer Step. Organometallics 1993, 12, 3390–3392. 10.1021/om00033a005.

[ref10] Foley B. L.; Johnson B. A.; Bhan A. A Method for Assessing Catalyst Deactivation: A Case Study on Methanol-to-Hydrocarbons Conversion. ACS Catal. 2019, 9, 7065–7072. 10.1021/acscatal.9b01106.

[ref11] Vu B. K.; Song M. B.; Park S.-A.; Lee Y.; Ahn I. Y.; Suh Y.-W.; Suh D. J.; Kim W.-I.; Koh H.-L.; Choi Y. G.; Shin E. W. Electronic Density Enrichment of Pt Catalysts by Coke in The Propane Dehydrogenation. Korean J. Chem. Eng. 2011, 28, 383–387. 10.1007/s11814-010-0363-8.

[ref12] Nørskov J. K.; Abild-Pedersen F.; Studt F.; Bligaard T. Density Functional Theory in Surface Chemistry and Catalysis. Proc. Natl. Acad. Sci. U.S.A. 2011, 108, 937 10.1073/pnas.1006652108. 21220337 PMC3024687

[ref13] Abild-Pedersen F.; Greeley J.; Studt F.; Rossmeisl J.; Munter T. R.; Moses P. G.; Skúlason E.; Bligaard T.; Nørskov J. K. Scaling Properties of Adsorption Energies for Hydrogen-Containing Molecules on Transition-Metal Surfaces. Phys. Rev. Lett. 2007, 99, 016105 10.1103/PhysRevLett.99.016105. 17678168

[ref14] Greeley J.; Nørskov J. K.; Mavrikakis M. Electronic structure and catalysis on metal surfaces. Annu. Rev. Phys. Chem. 2002, 53, 319–348. 10.1146/annurev.physchem.53.100301.131630. 11972011

[ref15] Lausche A. C.; Hummelshøj J. S.; Abild-Pedersen F.; Studt F.; Nørskov J. K. Application of A New Informatics Tool in Heterogeneous Catalysis: Analysis of Methanol Dehydrogenation on Transition Metal Catalysts for The Production of Anhydrous Formaldehyde. J. Catal. 2012, 291, 133–137. 10.1016/j.jcat.2012.04.017.

[ref16] Wang S.; Petzold V.; Tripkovic V.; Kleis J.; Howalt J. G.; Skúlason E.; Fernández E. M.; Hvolbæk B.; Jones G.; Toftelund A.; Falsig H.; Björketun M.; Studt F.; Abild-Pedersen F.; Rossmeisl J.; Nørskov J. K.; Bligaard T. Universal Transition State Scaling Relations for (De)Hydrogenation over Transition Metals. Phys. Chem. Chem. Phys. 2011, 13, 20760–20765. 10.1039/C1CP20547A. 21996683

[ref17] Hammer B.; Nørskov J. K. Theoretical surface science and catalysis—calculations and concepts. In Impact of Surface Science on Catalysis; Academic Press: 2000; Vol. 45, pp 71–129.

[ref18] Hansen M. H.; Nørskov J. K.; Bligaard T. First Principles Micro-Kinetic Model of Catalytic Non-Oxidative Dehydrogenation of Ethane over Close-Packed Metallic Facets. J. Catal. 2019, 374, 161–170. 10.1016/j.jcat.2019.03.034.

[ref19] Nykänen L.; Honkala K. Density Functional Theory Study on Propane and Propene Adsorption on Pt(111) and PtSn Alloy Surfaces. J. Phys. Chem. C 2011, 115, 9578–9586. 10.1021/jp1121799.

[ref20] Raman N.; Maisel S.; Grabau M.; Taccardi N.; Debuschewitz J.; Wolf M.; Wittkämper H.; Bauer T.; Wu M.; Haumann M.; Papp C.; Görling A.; Spiecker E.; Libuda J.; Steinrück H.-P.; Wasserscheid P. Highly Effective Propane Dehydrogenation Using Ga–Rh Supported Catalytically Active Liquid Metal Solutions. ACS Catal. 2019, 9, 9499–9507. 10.1021/acscatal.9b02459. 32219008 PMC7088128

[ref21] Raman N.; Wolf M.; Heller M.; Heene-Würl N.; Taccardi N.; Haumann M.; Felfer P.; Wasserscheid P. GaPt Supported Catalytically Active Liquid Metal Solution Catalysis for Propane Dehydrogenation–Support Influence and Coking Studies. ACS Catal. 2021, 11, 13423–13433. 10.1021/acscatal.1c01924. 34777909 PMC8576810

[ref22] Taccardi N.; Grabau M.; Debuschewitz J.; Distaso M.; Brandl M.; Hock R.; Maier F.; Papp C.; Erhard J.; Neiss C.; Peukert W.; Görling A.; Steinrück H. P.; Wasserscheid P. Gallium-Rich Pd–Ga Phases as Supported Liquid Metal Catalysts. Nat. Chem. 2017, 9, 862–867. 10.1038/nchem.2822. 28837180

[ref23] Sebastian O.; Nair S.; Taccardi N.; Wolf M.; Søgaard A.; Haumann M.; Wasserscheid P. Stable and Selective Dehydrogenation of Methylcyclohexane using Supported Catalytically Active Liquid Metal Solutions – Ga52Pt/SiO2 SCALMS. ChemCatChem 2020, 12, 4533–4537. 10.1002/cctc.202000671.

[ref24] Wolf M.; Raman N.; Taccardi N.; Haumann M.; Wasserscheid P. Coke Formation during Propane Dehydrogenation over Ga–Rh Supported Catalytically Active Liquid Metal Solutions. ChemCatChem 2020, 12, 1085–1094. 10.1002/cctc.201901922. 32194874 PMC7074060

[ref25] Wittkämper H.; Hock R.; Weißer M.; Dallmann J.; Vogel C.; Raman N.; Tacardi N.; Haumann M.; Wasserscheid P.; Hsieh T.-E.; Maisel S.; Moritz M.; Wichmann C.; Frisch J.; Gorgoi M.; Wilks R. G.; Bär M.; Wu M.; Spiecker E.; Görling A.; Unruh T.; Steinrück H.-P.; Papp C. Isolated Rh Atoms in Dehydrogenation Catalysis. Sci. Rep. 2023, 13, 4458 10.1038/s41598-023-31157-y. 36932106 PMC10023779

[ref26] Wittkämper H.; Maisel S.; Wu M.; Frisch J.; Wilks R. G.; Grabau M.; Spiecker E.; Bär M.; Görling A.; Steinrück H.-P.; Papp C. Oxidation Induced Restructuring of Rh–Ga SCALMS Model Catalyst Systems. J. Chem. Phys. 2020, 153, 104702 10.1063/5.0021647. 32933289

[ref27] Hsieh T.-E.; Maisel S.; Wittkämper H.; Frisch J.; Steffen J.; Wilks R. G.; Papp C.; Görling A.; Bär M. Unraveling the Effect of Rh Isolation on Shallow d States of Gallium–Rhodium Alloys. J. Phys. Chem. C 2023, 127, 20484–20490. 10.1021/acs.jpcc.3c04350.

[ref28] Davison S. G.; Sulston K. W. Anderson-Newns-Grimley Model. In Green-Function Theory of Chemisorption; Davison S. G.; Sulston K. W., Eds.; Springer Netherlands: Dordrecht, 2006; pp 45–74.

[ref29] Greiner M. T.; Jones T. E.; Beeg S.; Zwiener L.; Scherzer M.; Girgsdies F.; Piccinin S.; Armbrüster M.; Knop-Gericke A.; Schlögl R. Free-Atom-Like d States in Single-Atom Alloy Catalysts. Nat. Chem. 2018, 10, 1008–1015. 10.1038/s41557-018-0125-5. 30150725

[ref30] Carli R.; Bianchi C. L. XPS Analysis of Gallium Oxides. Appl. Surf. Sci. 1994, 74, 99–102. 10.1016/0169-4332(94)90104-X.

[ref31] Chabala J. M. Oxide-Growth Kinetics and Fractal-Like Patterning Across Liquid Gallium Surfaces. Phys. Rev. B 1992, 46, 11346–11357. 10.1103/PhysRevB.46.11346. 10003021

[ref32] Fouquat L.; Vettori M.; Botella C.; Benamrouche A.; Penuelas J.; Grenet G. X-ray Photoelectron Spectroscopy Study of Ga Nanodroplet on Silica-Terminated Silicon Surface for Nanowire Growth. J. Cryst. Growth 2019, 514, 83–88. 10.1016/j.jcrysgro.2019.03.003.

[ref33] Butt D. P.; Park Y.; Taylor T. N. Thermal Vaporization and Deposition of Gallium Oxide in Hydrogen. J. Nucl. Mater. 1999, 264, 71–77. 10.1016/S0022-3115(98)00484-X.

[ref34] Lee S. W.; Subramanian A.; Zamudio F. B.; Zhong J. Q.; Kozlov S. M.; Shaikhutdinov S.; Roldan Cuenya B. Interaction of Gallium with a Copper Surface: Surface Alloying and Formation of Ordered Structures. J. Phys. Chem. C 2023, 127, 20700–20709. 10.1021/acs.jpcc.3c05711. PMC10614298 37908742

[ref35] Lee S. W.; Luna M. L.; Berdunov N.; Wan W.; Kunze S.; Shaikhutdinov S.; Cuenya B. R. Unraveling Surface Structures of Gallium Promoted Transition Metal Catalysts in CO2 Hydrogenation. Nat. Commun. 2023, 14, 4649 10.1038/s41467-023-40361-3. 37532720 PMC10397205

[ref36] Zinkevich M.; Aldinger F. Thermodynamic Assessment of the Gallium-Oxygen. System 2004, 87, 683–691. 10.1111/j.1551-2916.2004.00683.x.

[ref37] Lips K.; Starr D. E.; Bär M.; Schulze T. F.; Fenske F.; Christiansen S.; Krol R. v. d.; Raoux S.; Reichardt G.; Schäfers F.; Hendel S.; Follath R.; Bahrdt J.; Scheer M.; Wüstefeld G.; Kuske P.; Hävecker M.; Knop-Gericke A.; Schlögl R.; Rech B. EMIL: The Energy Materials In Situ Laboratory Berlin; Proc. 40th IEEE Photovoltaic Specialists Conference, 2014, 698.

[ref38] Shinotsuka H.; Tanuma S.; Powell C. J.; Penn D. R. Calculations of Electron Inelastic Mean Free Paths. XII. Data for 42 Inorganic Compounds over The 50 eV to 200 keV Range with The Full Penn Algorithm. Surf. Interface Anal. 2019, 51, 427–457. 10.1002/sia.6598. PMC7047655 32116395

[ref39] Shinotsuka H.; Da B.; Tanuma S.; Yoshikawa H.; Powell C. J.; Penn D. R. Calculations of Electron Inelastic Mean Free Paths. XI. Data for Liquid Water for Energies from 50 eV to 30 keV. Surf. Interface Anal. 2017, 49, 238–252. 10.1002/sia.6123. 28751796 PMC5524379

[ref40] Shinotsuka H.; Tanuma S.; Powell C. J.; Penn D. R. Calculations of Electron Inelastic Mean Free Paths. X. Data for 41 Elemental Solids over The 50 eV to 200 keV Range with The Relativistic Full Penn Algorithm. Surf. Interface Anal. 2015, 47, 1132–1132. 10.1002/sia.5861. PMC7047655 32116395

[ref41] Seah M. P.; Dench W. A. Quantitative Electron Spectroscopy of Surfaces: A Standard Data Base for Electron Inelastic Mean Free Paths in Solids. Surf. Interface Anal. 1979, 1, 2–11. 10.1002/sia.740010103.

[ref42] Hsieh T.-E.; Frisch J.; Wilks R. G.; Bär M. Unravelling the Surface Oxidation-Induced Evolution of the Electronic Structure of Gallium. ACS Appl. Mater. Interfaces 2023, 15, 47725–47732. 10.1021/acsami.3c09324. 37774118 PMC10571040

[ref43] Grabau M.; Krick Calderón S.; Rietzler F.; Niedermaier I.; Taccardi N.; Wasserscheid P.; Maier F.; Steinrück H.-P.; Papp C. Surface Enrichment of Pt in Ga2O3 Films Grown on Liquid Pt/Ga Alloys. Surf. Sci. 2016, 651, 16–21. 10.1016/j.susc.2016.03.009.

[ref44] Wittkämper H.; Maisel S.; Moritz M.; Grabau M.; Görling A.; Steinrück H. P.; Papp C. Surface Oxidation-induced Restructuring of Liquid Pd-Ga SCALMS Model Catalysts. Phys. Chem. Chem. Phys. 2021, 23, 16324–16333. 10.1039/D1CP02458B. 34313278

[ref45] Kibis L. S.; Stadnichenko A. I.; Koscheev S. V.; Zaikovskii V. I.; Boronin A. I. XPS Study of Nanostructured Rhodium Oxide Film Comprising Rh4+ Species. J. Phys. Chem. C 2016, 120, 19142–19150. 10.1021/acs.jpcc.6b05219.

[ref46] Pearton S. J.; Yang J.; Cary P. H.; Ren F.; Kim J.; Tadjer M. J.; Mastro M. A. A Review of Ga2O3 Materials, Processing, and Devices. Appl. Phys. Rev. 2018, 5, 011301 10.1063/1.5006941.

[ref47] Niu H.; Bonati L.; Piaggi P. M.; Parrinello M. Ab initio phase diagram and nucleation of gallium. Nat. Commun. 2020, 11, 2654 10.1038/s41467-020-16372-9. 32461573 PMC7253470

[ref48] Anres P.; Gaune-Escard M.; Bros J. P. Thermodynamics of the (Rh–Ga) System. J. Alloys Compd. 1998, 265, 201–208. 10.1016/S0925-8388(97)00420-9.

[ref49] Cochran C. N.; Foster L. M. Vapor Pressure of Gallium, Stability of Gallium Suboxide Vapor, and Equilibria of Some Reactions Producing Gallium Suboxide Vapor. J. Electrochem. Soc. 1962, 109, 144 10.1149/1.2425347.

[ref50] Ohira S.; Arai N.; Oshima T.; Fujita S. Atomically Controlled Surfaces with Step and Terrace of β-Ga2O3 Single Crystal Substrates for Thin Film Growth. Appl. Surf. Sci. 2008, 254, 7838–7842. 10.1016/j.apsusc.2008.02.184.

[ref51] Williams C. T.; Chen K. Y.; Takoudis C. G.; Weaver M. J. Reduction Kinetics of Surface Rhodium Oxide by Hydrogen and Carbon Monoxide at Ambient Gas Pressures As Probed by Transient Surface-Enhanced Raman Spectroscopy. J. Phys. Chem. B 1998, 102, 4785–4794. 10.1021/jp981287d.

[ref52] Kellogg G. L. Oxide Formation and Reduction on Rhodium Surfaces. Surf. Sci. 1986, 171, 359–376. 10.1016/0039-6028(86)91086-1.

[ref53] Toyoshima R.; Ueda K.; Koda Y.; Kodama H.; Sumida H.; Mase K.; Kondoh H. In situ AP-XPS Study on Reduction of Oxidized Rh Catalysts under CO Exposure and Catalytic Reaction Conditions. J. Phys. D: Appl. Phys. 2021, 54, 204005 10.1088/1361-6463/abe486.

[ref54] Hohner C.; Kettner M.; Stumm C.; Blaumeiser D.; Wittkämper H.; Grabau M.; Schwarz M.; Schuschke C.; Lykhach Y.; Papp C.; Steinrück H.-P.; Libuda J. Pt–Ga Model SCALMS on Modified HOPG: Thermal Behavior and Stability in UHV and under Near-Ambient Conditions. J. Phys. Chem. C 2020, 124, 2562–2573. 10.1021/acs.jpcc.9b10944.

[ref55] Yamada T.; Terashima D.; Nozaki M.; Yamada H.; Takahashi T.; Shimizu M.; Yoshigoe A.; Hosoi T.; Shimura T.; Watanabe H. Controlled Oxide Interlayer for Improving Reliability of SiO2/GaN MOS Devices. Jpn. J. Appl. Phys. 2019, 58, SCCD06 10.7567/1347-4065/ab09e0.

