# Supplementary material for: Impact of Catalysis-Relevant Oxidation and Annealing Treatments on Nanostructured GaRh Alloys
Source: ACS Appl Mater Interfaces. 2024 Apr 9;16(15):19858–65. doi: 10.1021/acsami.4c02286 (PMC11040576; doi:10.1021/acsami.4c02286)
Supplement: Supplementary file 1 — am4c02286_si_001.pdf [file am4c02286_si_001.pdf]

Supporting Information

Impact of Catalysis-Relevant Oxidation and Annealing Treatments on Nanostructured GaRh Alloys

Tzung-En Hsieh[1*], Johannes Frisch[1,3], Regan G. Wilks[1,3], Christian Papp[5], Marcus Bär[1,2,3,4*]

[1]*Department Interface Design, Helmholtz-Zentrum Berlin für Materialien und Energie GmbH (HZB), 12489 Berlin, Germany*

[2]*Department of Chemistry and Pharmacy, Friedrich-Alexander-Universität Erlangen-Nürnberg (FAU), 91058 Erlangen, Germany*

[3]*Energy Materials In-situ Laboratory Berlin (EMIL), HZB, 12489 Berlin, Germany*

[4]*Department X-ray Spectroscopy at Interfaces of Thin Films, Helmholtz-Institute Erlangen-Nürnberg for Renewable Energy (HI ERN), 12489 Berlin, Germany*

[5]*Freie Universität Berlin, Physical and Theoretical Chemistry, 14195, Berlin, Germany*

Corresponding authors:

Tzung-En Hsieh: tzung-en.hsieh@helmholtz-berlin.de

Marcus Bär: marcus.baer@helmholtz-berlin.de

Table of Contents:

XPS analysis S3

1.1 Quantification of XPS data S3

1.2 Determination of Rh concentration and GaO_x/Ga ratio S4

1.3 GaO_x thickness investigation S5

Figure S1. UPS spectra of the nanostructured GaRh alloy after surface oxidation and annealing to 600°C.. S6

Figure S2. XPS spectra of nanostructured GaRh alloys after surface oxidation.............................. S7

Figure S3. SEM of oxidized nanostructured GaRh alloys before and after annealing S8

Figure S4. Size distribution of oxidized nanostructured GaRh particles on SiO_x/Si support S8

Figure S5. Fits of the Ga 3d XPS spectra of nanostructured GaRh alloys after oxidation S9

Figure S6. Fits of the Ga 2p XPS spectra of nanostructured GaRh alloys after oxidation S10

Figure S7. Fits of the Rh 3d XPS spectra of nanostructured GaRh alloys after oxidation S11

Figure S8. Ga feature subtracted He II-UPS spectra of nanostructured GaRh alloys after oxidation.... S12

Figure S9. Fits of the Ga $2p_{3/2}$ spectra of nanostructured GaRh alloys upon annealing S13

Figure S10. Fits of the Rh $3d_{5/2}$ spectra of the nanostructured GaRh alloys upon annealin S14

Figure S11. C 1s/Ga LMM spectra of the as-prepared nanostructured GaRh alloy sample at room temperature and at 650 °C S15

Figure S12. UPS spectra of GaRh alloys after oxidation and upon annealing.............................. S16

Figure S13. XPS spectra of the silicon wafer before and after Ar^+-ion sputtering......................... S17

Figure S14. Fits of the Ga $2p_{3/2}$ XPS spectra of the nanostructured GaRh alloy after 600°C annealing on SiO_x/Si support S18

Figure S15. SEM images of the nanostructured GaRh alloy deposited on SiO_x/Si and Si supports after annealing at 600 °C S19

Table S1. GaO_x thickness change upon oxidation derived from Ga3d fit results S20

Table S2. GaO_x thickness change upon oxidation derived from Ga2p$_{3/2}$ fit results S20

Table S3. Evolution of Rh concentration after oxidation derived from Ga 3d and Rh 3d fits S21

Table S4. Evolution of Rh concentration after oxidation derived from Ga 2p and Rh 3d fits......... S22

Table S5. Evolution of Rh concentration upon annealing derived from Ga 2p and Rh 3d fits S22

Table S6. GaO_x thickness change upon annealing derived from Ga2p fit results S22

References S23

XPS ANALYSIS

The total energy resolution of the XPS and UPS setup is determined via a fitting of the measured Fermi-edge (E_F) of a clean gold film by the following fit function with correction of temperature (T = 300 K) employing the Boltzmann constant ($k_b \cdot T$ = 25 meV):

$$f(x) = \frac{ax+b}{2} \times [1 - \mathrm{erf}\left(\frac{E_f - x}{\sigma_{total}\sqrt{2}}\right)] + cx + d$$

$$\sigma_{total} = \sqrt{(1.7 \times k_b \cdot T)^2 + \sigma^2}$$

$$exp.uncertainty = 2 \times \sqrt{2 \cdot ln2} \times \sigma$$

σ_{total} is the total Gaussian broadening including instrumental and thermal ($k_b \cdot T$) broadening, E_f denotes the energy of Fermi-edge. a, b, c, d are dependent variables in the fit function.

1.1 Quantification of XPS data

All XPS data were fitted and quantified by Winspec (LISE, Université de Paix, Namur). The XPS peaks of metallic components (GaRh IMCs, isolated Rh atoms, metallic Ga matrix) are fitted by an asymmetric (Doniach-Sunjic) profile; the XPS peaks ascribed to GaO_x are fitted by Voigt profiles. The fitting includes a Shirley background. In the Ga $2p_{3/2}$ and 3d fitting, a GaO_x peak with different peak width is used (Fig. S5, S6, S9 and S14) due to presence of oxygen vacancies[1] and several GaO_x species causing a peak broadening. The fits shown in Fig. S9 and S14 are conducted with the following constraints to obtain a reasonable fitting result for quantitative analysis. The peak width and position and intensity ratio of spin-orbit split doublet peaks are coupled in all fitting routines and the spectrum is fitted sequentially with fixed peak shape and width. The derived area of the studied core level peaks is corrected by the transmission function of the used analyzer:

$$I = I_0 * (0.61 + 0.00021 * (E_x - E_b)$$

I and I_0 denote the calibrated peak area and original peak area, respectively. E_x denotes the excitation energy (Mg K_α = 1253.56 eV), BE denotes to the binding energy of core level peak.

Additionally, the derived peak areas are corrected for by IMFP and photoionization cross sections. The IMFP value is derived by the TPP-2M formula.[2-4] Photoionization cross sections (σ) are calculated from the tabulated value from Trzhaskovskaya, Nefedov, and Yarzhemski,[5-6] considering the geometry of the XPS setup. The quantitative analyses of certain core level peaks or elements in this study are then processed by following equation:

$$\frac{A}{B} = \left(\frac{I_A}{\sigma_A} \div \frac{I_B}{\sigma_B} \div \frac{\mathrm{IMFP}_A}{\mathrm{IMFP}_B}\right)$$

I_A and I_B denote the peak area of core level A and B. σ_A and σ_B are the respective photoionization cross sections of core level A and B. $IMFP_A$ and $IMFP_B$ denote to inelastic mean free path of photoelectrons of core level A and B, respectively.

1.2 Determination of Rh concentration and GaO_x/Ga ratio

The fit results of Ga 3d, Ga $2p_{3/2}$, and Rh $3d_{5/2}$ core level peaks shown in Fig. S5, S6, S7, S9, S10 have been used to determine the Rh concentration of the GaRh sample before and after all oxidation steps. Note that for this consideration always the total peak area is used. The applied photoionization cross section (σ) of the Ga $3d_{5/2}$, Ga $2p_{3/2}$, and Rh $3d_{5/2}$ core levels are 1.61, 45.7 and 19.2 (arbitrary units), respectively.[5-7] The inelastic mean free path (IMFP) of the Ga $3d_{5/2}$, Ga $2p_{3/2}$, and Rh $3d_{5/2}$ photoelectrons in GaRh alloy material is 20.9, 5.3, and 17.2 Å, respectively.[2-4, 8] The Rh concentration is then calculated by following equation:

$$[Rh] = \left(\frac{\left(\frac{Rh}{Ga}\right)}{1 + \left(\frac{Rh}{Ga}\right)}\right) \cdot 100\ at\ \%$$

The results are shown in Table S3-S5. Note that the Rh content derived from the Ga 3d and Rh $3d_{5/2}$ spectra is less error prone (in case the sample has a significant chemical structure profile) as the corresponding photoelectrons have very similar kinetic energies and thus comparable IMFPs. The Ga $2p_{3/2}$ photoelectrons have a significantly lower kinetic energy and thus lower IMFP. Hence, the Rh content derived from the Ga $2p_{3/2}$ and Rh $3d_{5/2}$ lines – due to their

different information depths and different background profile– has a larger experimental uncertainty. The fitting shown in Fig. S5, S6, S9 are utilized to derive the intensity ratio of Ga and GaO_x which is used for GaO_x film thickness calculation elaborated in section 1.3.

1.3 Determination of GaO_x film thickness

In this study, a simple overlayer model is utilized to get insights on the GaO_x layer formation on top of metallic Ga, assuming a mechanism of homogeneous, closed packed oxide film growth.[9] The following equation can be used to calculate the GaO_x film thickness, D:

$$\mathrm{D} = \lambda_{i,\mathrm{Ga}O_x} \cdot \ln\left[\frac{I_{i,\mathrm{GaO}_x} \cdot \lambda_{i,\mathrm{Ga}} \cdot \mathrm{N(Ga)_{Ga}}}{I_{i,Ga} \cdot \lambda_{i,\mathrm{GaO_x}} \cdot \mathrm{N(Ga)_{GaO_x}}} + 1\right]$$

$\lambda_{i,\,GaOx}$ and $\lambda_{i,Ga}$ are the IMFP values in GaO_x and metallic Ga, respectively for core level *i* (calculated using the TPP2-M formula[2-4] with the density and electron configuration of stoichiometric Ga_2O_3 as the absorbing layer – we considered this the best approximation available due to the lack of reliable parameters for GaO_x). The $\lambda_{i,GaOx}$ and $\lambda_{i,Ga}$ values for the Ga 3d photoelectrons are 20.9 and 27.2 Å, respectively; for the Ga $2p_{3/2}$ photoelectrons, we use 5.3 and 6.3 Å, respectively.[2-4] $I_{i,GaOx}$ and $I_{i,Ga}$ are the intensities (i.e., areas) of the GaO_x and Ga peak contributions, respectively, derived for the core level *i* (obtained from XPS data fitting, see Fig. S5, S6, S9). $N(Ga)_{GaOx}$ and $N(Ga)_{Ga}$ are the atomic densities of Ga in Ga_2O_3 (0.038 Atoms per cubic Å) and Ga (0.053 Atoms per cubic Å), respectively.[10] It is noted that the formula for D assumes a uniform bi-layer system with a closed capping oxide layer, and thus the discrepancies in the oxide thicknesses calculated using Ga $2p_{3/2}$ (Table S1) and using Ga 3d (Table S2) are presumably related to any deviation from this assumption (e.g., a different sample topography and/or an incomplete coverage of the Ga by the oxide capping layer), which will cause an underestimation of the derived layer thickness. As the oxide coverage of Ga increases, the results of the two calculations converge.

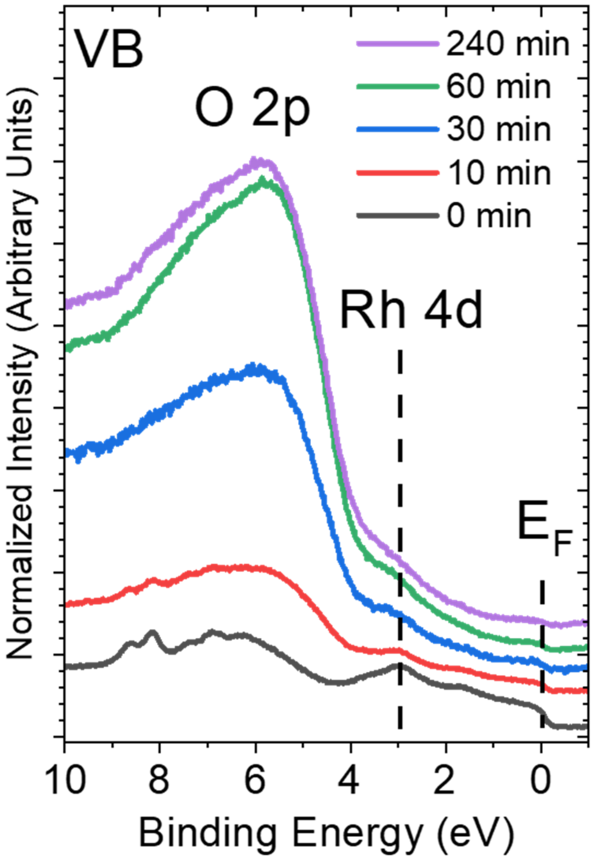

Figure S1. He II-UPS valence band spectra of the 7 at% Rh containing nanostructured GaRh alloy sample oxidized in 1×10^{-6} mbar O_2 for different times (0-240 min).

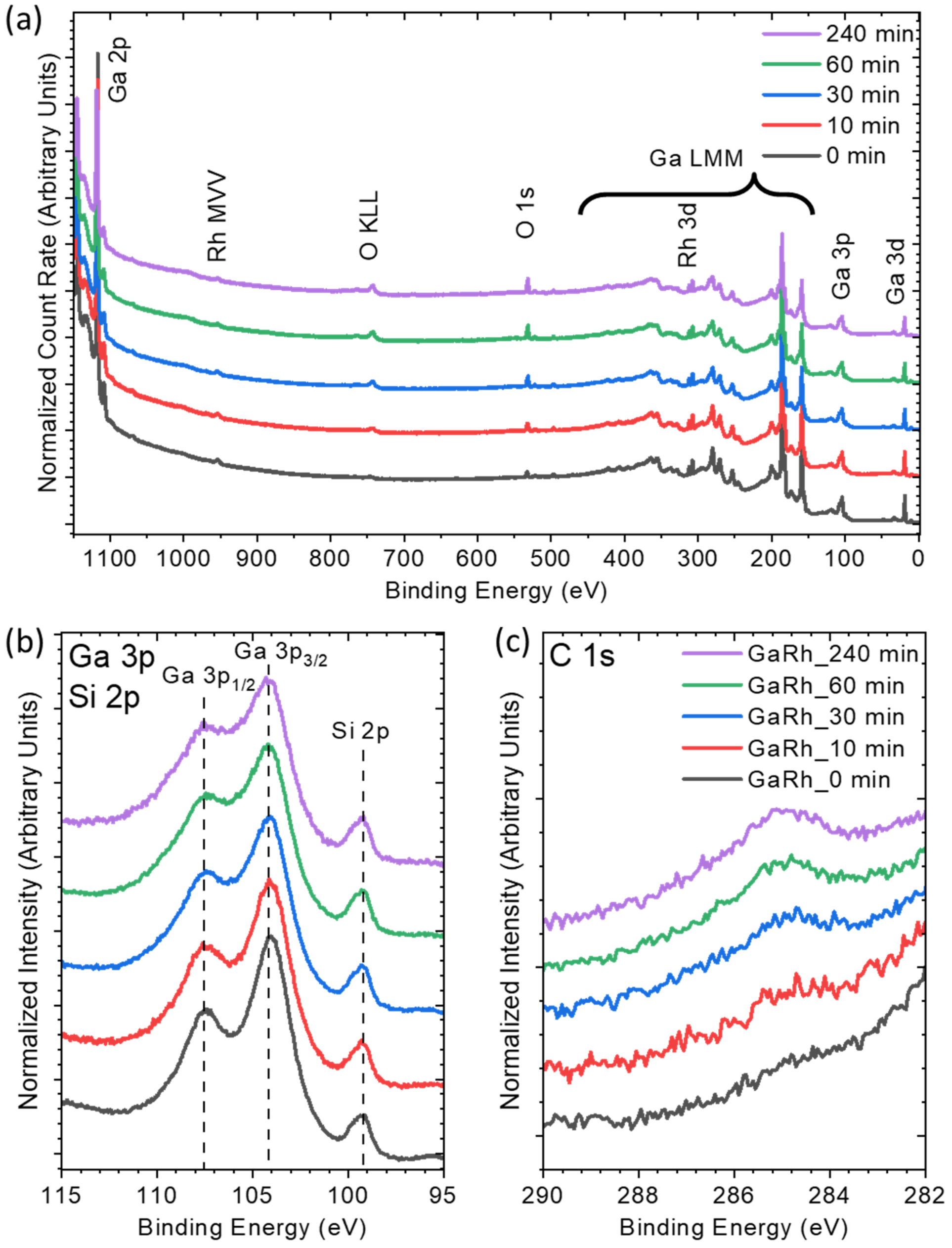

Figure S2. XPS (Mg K_α) survey (a), Ga 3p/Si 2p (b) and C 1s spectra (c) of the 7 at% Rh containing nanostructured GaRh alloys oxidized in 1×10^{-6} mbar O_2 for different times (0-240 min).

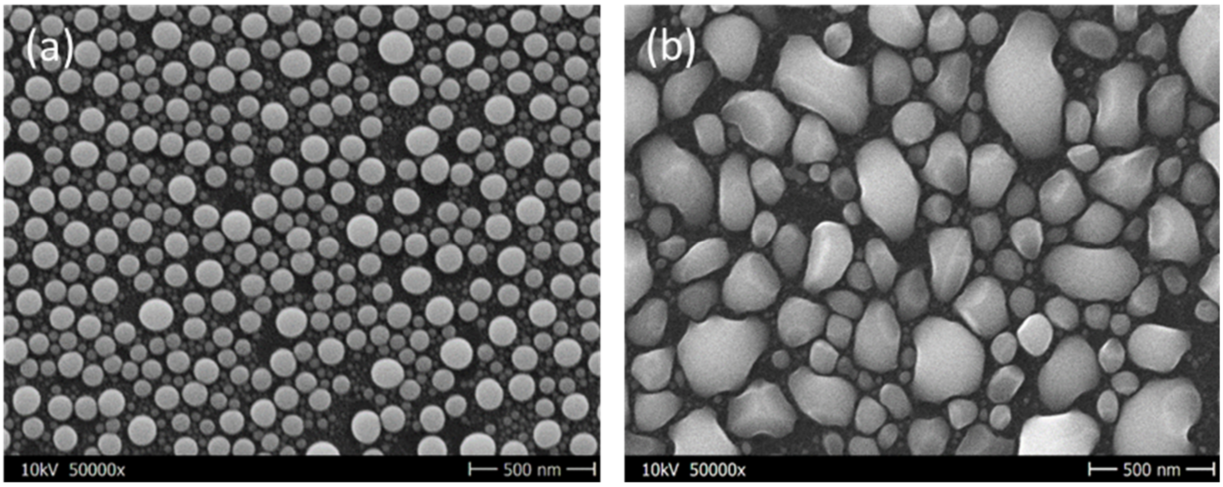

Figure S3. Representative SEM image of (initially oxidized) GaRh alloy samples with 7 at% Rh on SiO_x/Si support before (a) and after (b) 650 ºC annealing in vacuum conditions.

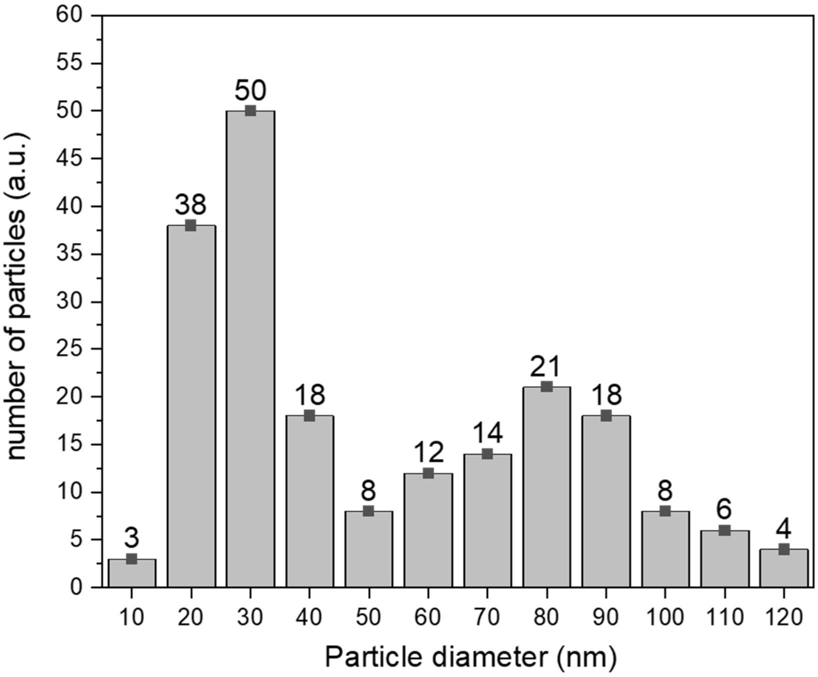

Figure S4. Analysis of the nanoparticle size distribution of the (initially oxidized) GaRh sample containing 7% Rh on SiO_x/Si support. The statistical analysis is based on 200 particles depicted from the SEM image shown in Fig. S3a.

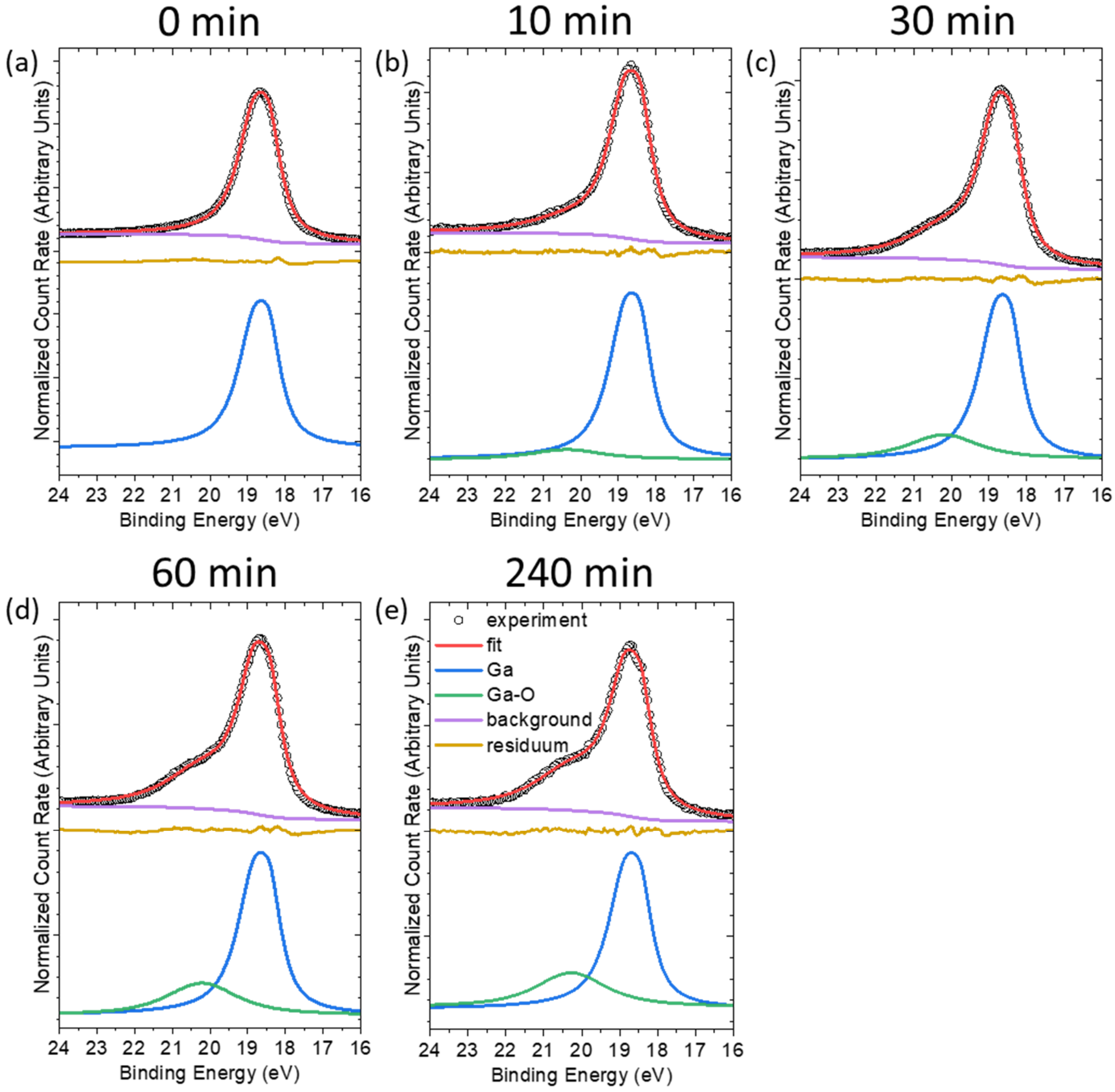

Figure S5. Fits of the Ga 3d XPS data collected with Mg K_α excitation for 7 at% Rh containing nanostructured GaRh samples oxidized in 1×10^{-6} mbar O_2 for (a) 0 min, (b) 10 min, (c) 30 min, (d) 60 min, and (e) 240 min. The metallic Ga peak is fitted by an asymmetric (Doniach-Sunjic) profile, and the GaO_x feature is fitted by a Voigt profile. The broader peak shape used to fit the GaO_x contribution is tentatively attributed to different oxide environments and/or due to the formed oxide being a less ordered material (compared to the metallic Ga) resulting in varying bond lengths and bond angles – all of which causes BE variations that may increase the FWHM of the Gaussian contribution of the Voigt profile used to fit this spectral component.

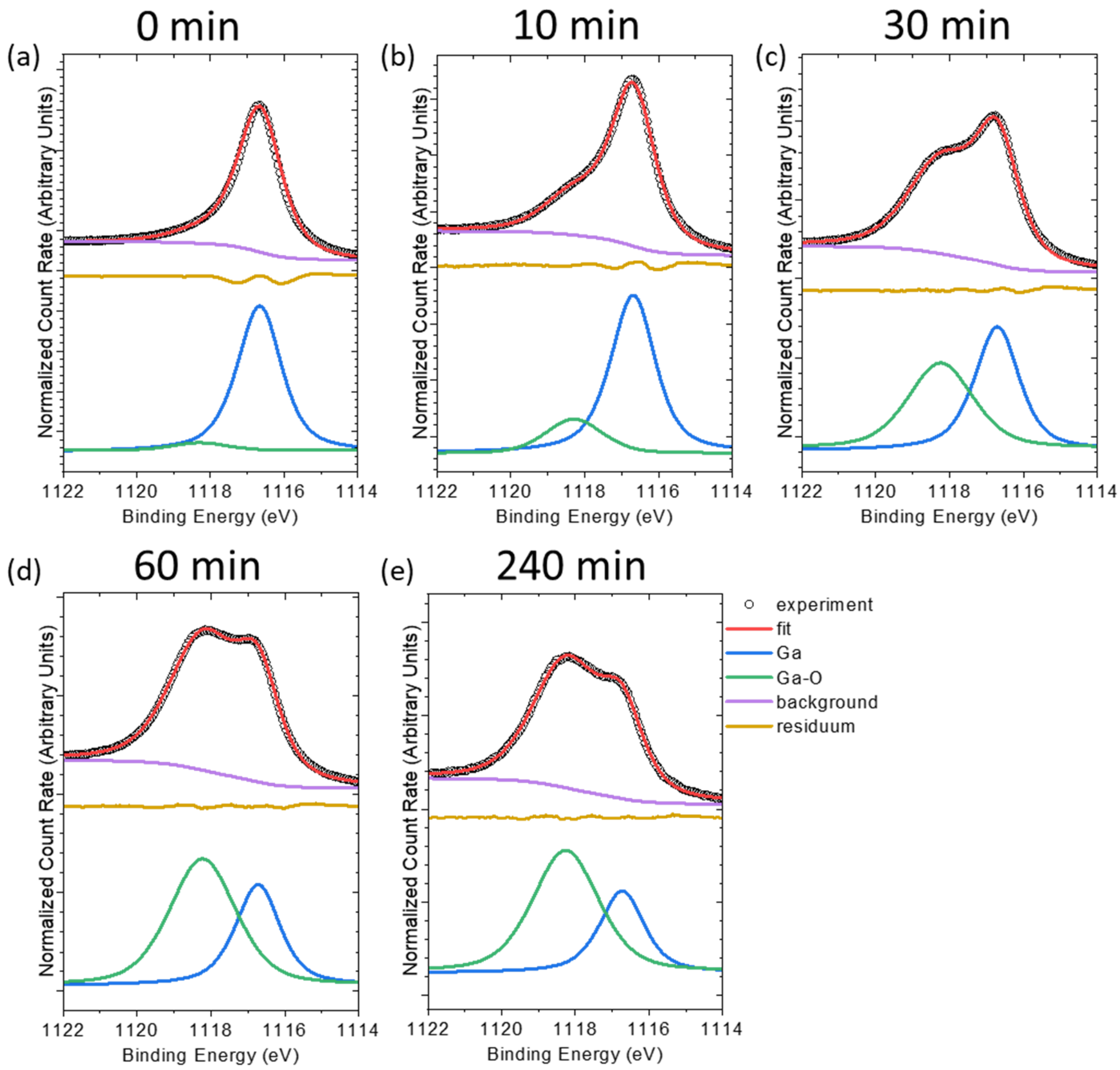

Figure S6. Fitting results of the Ga $2p_{3/2}$ XPS data collected with Mg K_α excitation for GaRh samples oxidized in 1×10^{-6} mbar O_2 for (a) 0 min, (b) 10 min, (c) 30 min, (d) 60 min, and (e) 240 min. The metallic Ga peak is fitted by an asymmetric (Doniach-Sunjic) profile, and the GaO_x feature is fitted by a Voigt profile. As discussed in the main text, a broad peak is used to encompass all present phases and compositions of GaO_x contribution with varying BE, rather than representing a single species at the specific binding energy defined by the Voigt function. This approach allows the GaO_x contribution to the spectra to be monitored, which is sufficient for the analysis contained here, but leads to imperfect fitting of the measured spectra. This is particularly apparent around 1117.7 eV in the 60- and 240-min oxidized sample, where an indication for a distinct spectral feature can be observed, which may be attributed to intrinsic (i.e., not n-type) Ga_2O_3.

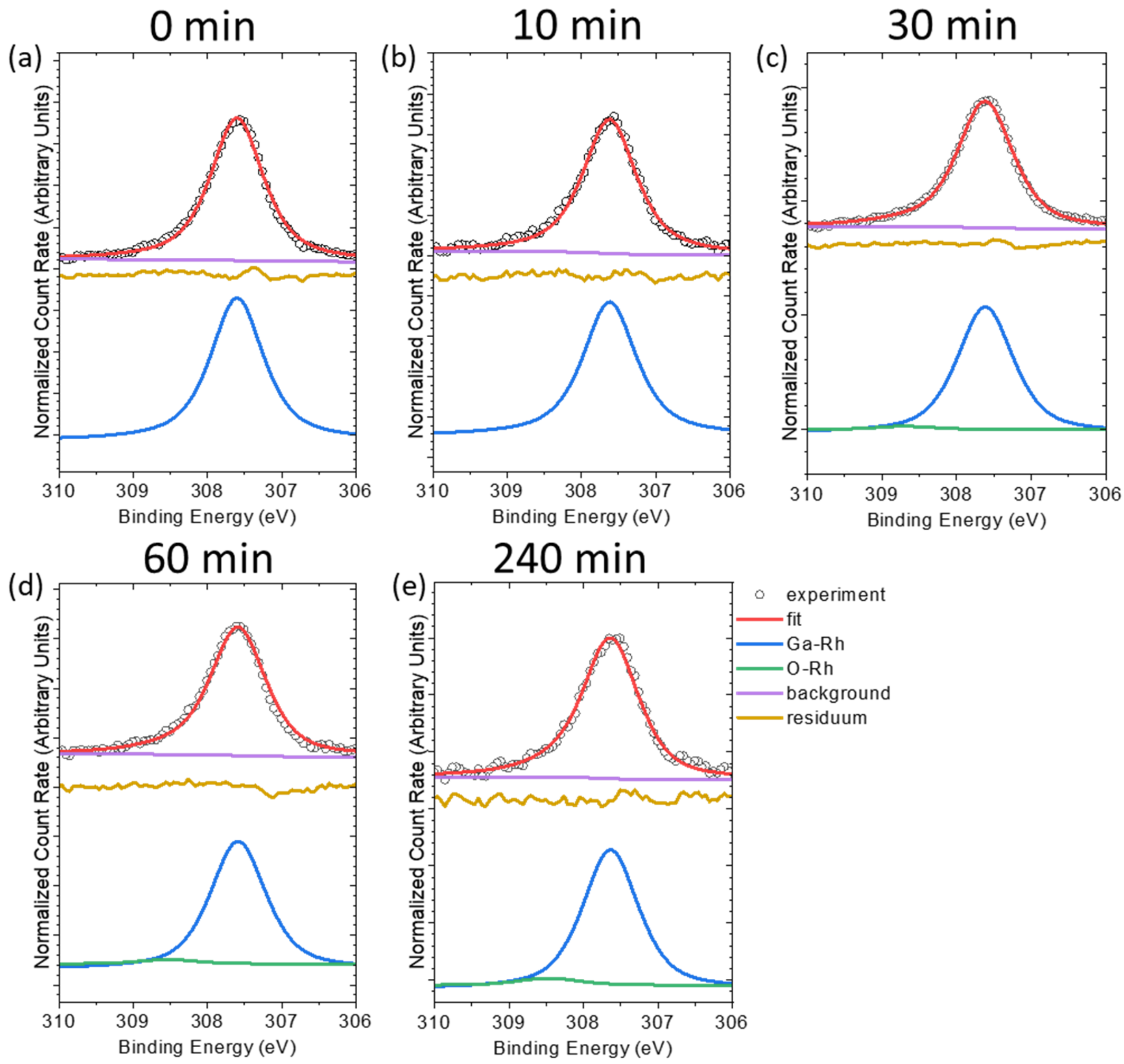

Figure S7. Fits of the Rh $3d_{5/2}$ XPS data collected with Mg K_α excitation for a 7 at% Rh containing nanostructured GaRh sample oxidized in 1×10^{-6} mbar O_2 for (a) 0 min, (b) 10 min, (c) 30 min, (d) 60 min, and (e) 240 min. The Ga-Rh peak is fitted by an asymmetric (Doniach-Sunjic) profile and O-Rh peak is fitted by Voigt profile. The minor Rh feature (green line) is ascribed to Rh atoms located in/close to the GaO_x layer (presumably forming Rh-O bonds). This contribution is fitted by a peak with broader FWHM which is tentatively attributed to a less ordered material (compared to the metallic GaRh alloy) resulting in varying bond lengths and bond angles – all of which causing BE variations that may increase the FWHM.

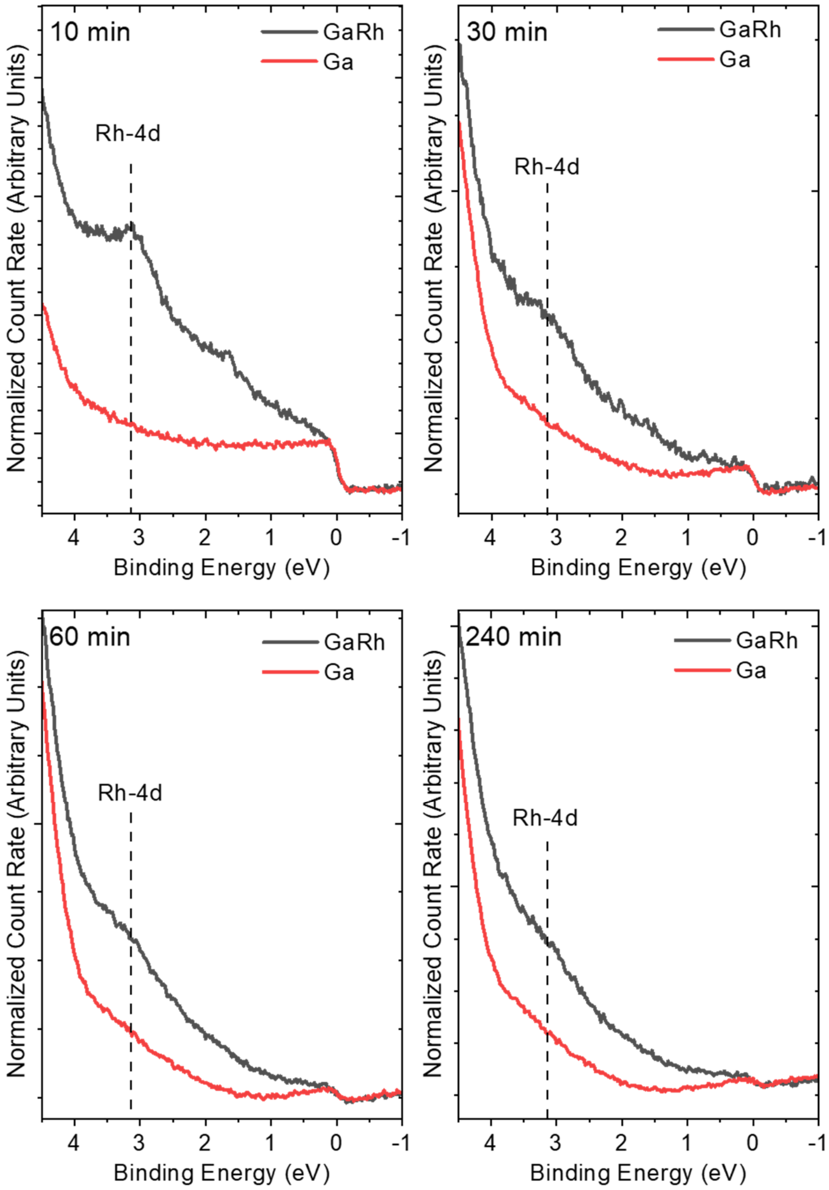

Figure S8. He II-UPS spectra of a 7 at% Rh containing nanostructured GaRh alloy compared to that of a pure (Rh-free) Ga reference sample (from Ref. 1) oxidized in 1×10^{-6} mbar O_2 for different times (10-240 min). The Ga spectrum is scaled to have a similar intensity of Fermi edge. The position of the Rh 4d derived spectral feature is indicated by the vertical dashed line. The spectral intensity around 3 eV in the Ga spectra is attributed to oxygen vacancies.[1]

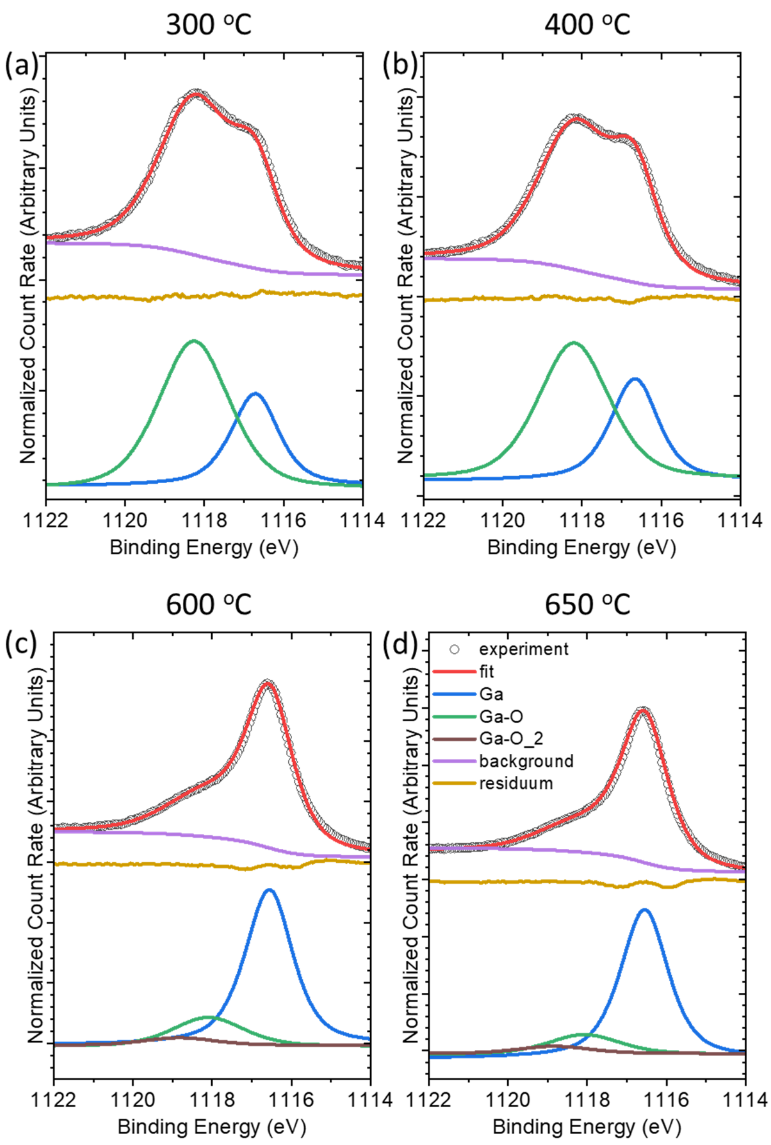

Figure S9. Fits of the Ga $2p_{3/2}$ XPS data collected with Mg K_α excitation for a 7 at% Rh containing nanostructured GaRh alloy sample annealed in vacuum conditions ($\leq 1\times10^{-8}$ mbar) at (a) 300 °C, (b) 400 °C, (c) 600 °C, and (d) 650 °C. Note a second Ga-O bond attributed spectral component (Ga-O_2) has to be included in the fit to obtain an acceptable fit result; this feature is attributed to a new GaO_x species formed during annealing process at 600 °C or above due to a substrate-induced oxidation. The metallic Ga peak is fitted by an asymmetric (Doniach-Sunjic) profile, and the GaO_x features are fitted by Voigt profiles. The broader peak shape used to fit the GaO_x contribution is tentatively attributed to different oxide environments and/or due to the formed oxide being a less ordered material (compared to the metallic Ga) resulting in varying bond lengths and bond angles – all of which causes BE variations that may increase the FWHM of the Gaussian contribution of the Voigt profile used to fit this spectral component.

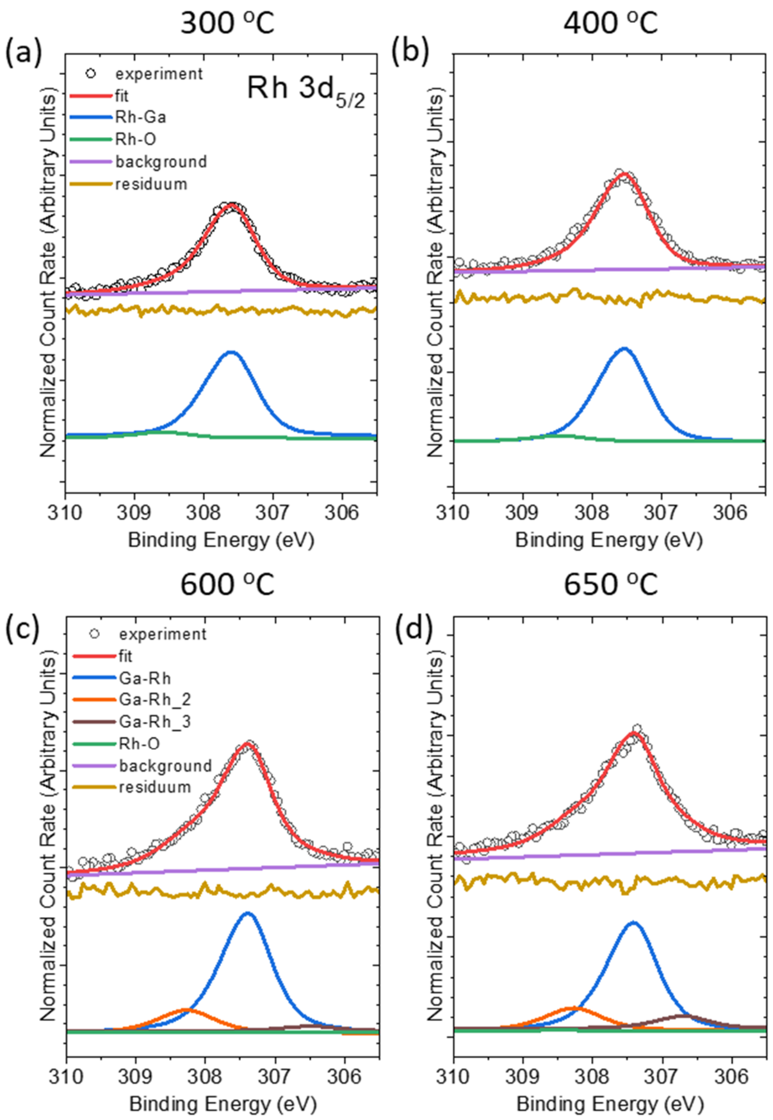

Figure S10. Fits of the Rh 3d$_{5/2}$ XPS data collected with Mg K_α excitation for a 7 at% Rh containing nanostructured GaRh alloy sample annealed in vacuum conditions ($\leq 1\times10^{-8}$ mbar) at (a) 300 ºC, (b) 400 ºC, (c) 600 ºC, and (d) 650 ºC. All Rh peaks are fitted by an asymmetric (Doniach-Sunjic) profile. The different peak width used in fitting is tentatively attributed to different chemical environments and/or due to the structural defects resulting in varying bond lengths and bond angles – all of which causing BE variations that may increase the FWHM. At low temperatures two fit components are sufficient for a good fit. The minor contribution ("Rh-O") is attributed to Rh-O bonds. At high temperatures two new spectral components have to be considered to result in a reasonable fit ("Gh-Rh_2" and Gh-Rh_3"). Note that Ga-Rh_2 has a different peak shape and position (308.3 eV) compared to the Rh-O peak (308.6 eV). See main text for detailed discussion.

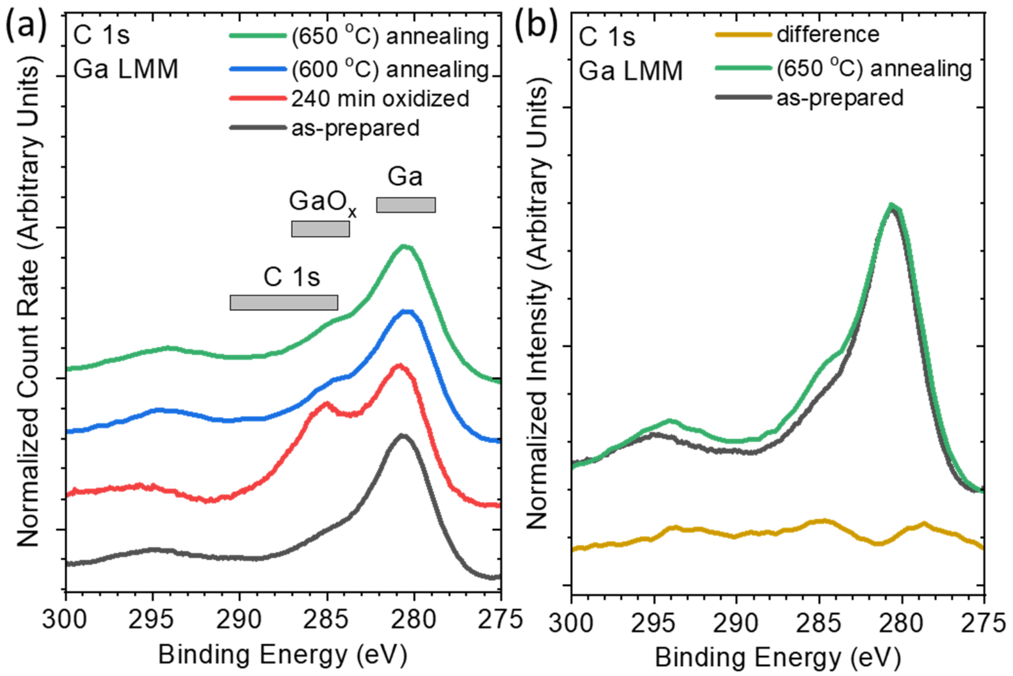

Figure S11. (a) Mg K_α-XPS C1s and Ga LMM Auger spectra of a 7 at% Rh containing GaRh alloy sample after different treatment steps. The gray boxes indicate reference BE positions for overlapping C 1s and Ga LMM related spectral features (taken from Ref. 1). (b) Direct comparison of the C 1s / Ga LMM spectra of (i) the as-prepared GaRh alloy sample and of (ii) the GaRh alloy sample measured at 650°C. The difference spectrum (referred to as "difference") shows (ii)-(i).

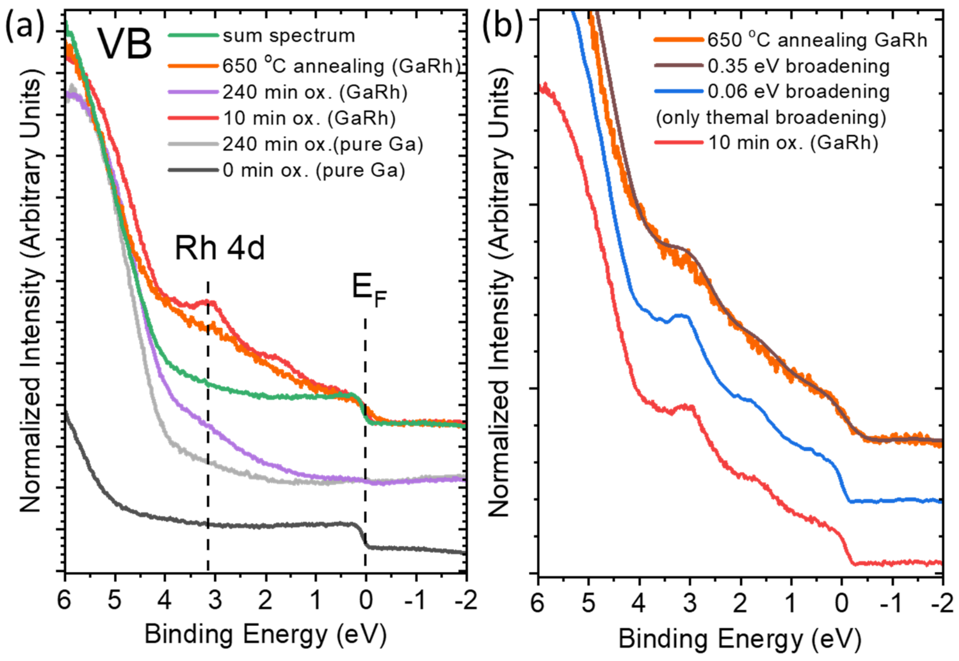

Figure S12. (a) He II-UPS spectra of 7 at% Rh containing nanostructured GaRh alloy samples oxidized in 1×10^{-6} mbar O_2 for 10 and 240 min (same spectra as in Fig. 1d) and the same sample measured at 650ºC (same spectrum as in Fig. 2c). The UPS spectra of an as-prepared pure Ga sample before and after oxidation in 1×10^{-6} mbar O_2 for 240 min (taken from Ref. 1) as well as the corresponding spectral sum of 50% of the VB spectrum of pure Ga before and 50% of the VB spectrum of the pure Ga after oxidation (referred to as "sum spectrum") are depicted for reference. The direct comparisons of the sum spectrum with that of the VB spectrum of the GaRh sample measured at 650°C on the one hand and the comparison of the spectra of the GaRh and pure Ga samples oxidized in 1×10^{-6} mbar O_2 for 240 min on the other hand emphasize the impact of the presence of Rh on the VB spectrum. (b) Comparison of the He II-UPS spectra of the 7 at% Rh containing nanostructure GaRh alloy sample collected at 650ºC and the spectrum of a similarly oxidized sample (1×10^{-6} mbar O_2 for 10 min). The blue and black spectra in (b) are derived from a convolution of the spectrum of the 7 at% Rh containing nanostructure GaRh alloy sample after 10 minutes oxidation (red) with a 0.06 and 0.35 eV Gaussian broadening. 0.06 eV is the expected thermal broadening, however, a broadening of 0.35 eV is required to match the even more broadened Fermi-edge feature at 0 eV of the actual measurement performed at 650°C, respectively. We attribute this discrepancy to the presence of multiple species with spectral intensity in this region. The spectra in (a) except that of pure Ga are normalized to the spectral intensity at 6 eV. The spectra in (b) are normalized to the Fermi-edge height at 0 eV and then stacked for clarity.

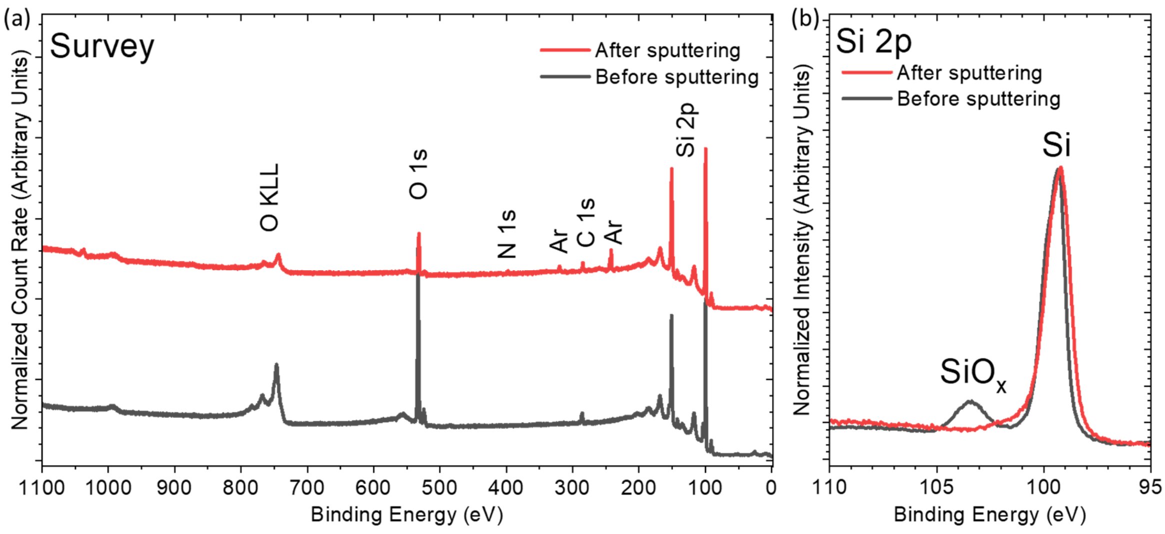

Figure S13. Mg K_α-XPS (a) survey spectra and (b) Si 2p spectra of the Si support before and after the removal of the native SiO_x by Ar^+-ion sputtering.

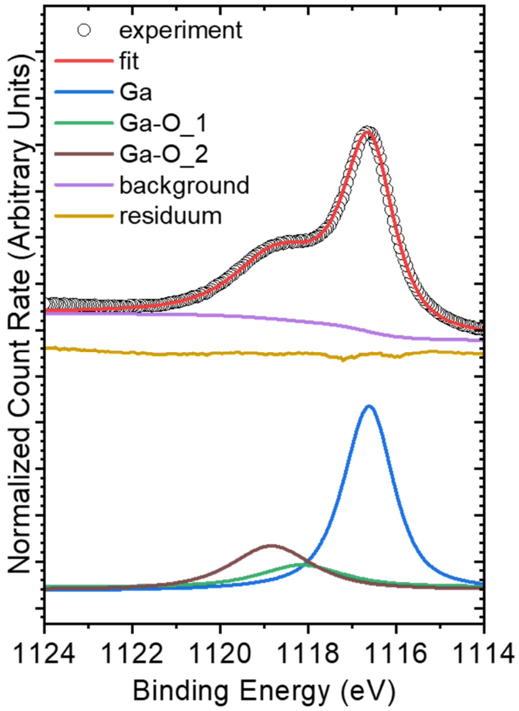

Figure S14. Fit of the Mg K_{α} Ga $2p_{3/2}$ XPS data of a 7 at% Rh containing nanostructured GaRh sample on SiO_x/Si support annealed at 600°C for 30 minutes in vacuum conditions ($\leq 1\times10^{-8}$ mbar). The sample is annealed directly after GaRh deposition and initial XPS characterization. The metallic Ga peaks are fitted by an asymmetric (Doniach-Sunjic) profile. Two GaO_x peaks (referred to as "Ga-O_1" and "Ga-O_2") are fitted by Voigt profiles. The different peak width used in fitting is tentatively attributed to different chemical environments and/or due to the structural defects resulting in varying bond lengths and bond angles – all of which causing BE variations that may increase the FWHM of the Gaussian contribution of the Voigt profile used to fit this spectral component.

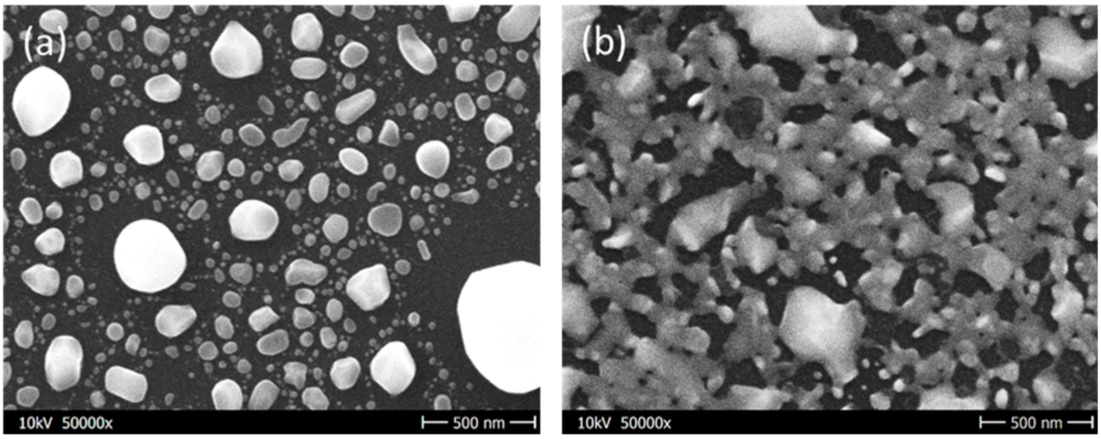

Figure S15. Representative SEM images of GaRh alloy samples with 7 at% Rh deposited on (a) SiO_x/Si support and (b) sputter-cleaned Si support after vacuum ($\leq 1\times10^{-8}$ mbar) annealing to 600°C for 30 min.

Table S1. GaO_x/metallic Ga ratio of the respective spectral contributions to the Ga $2p_{3/2}$ core level and calculated GaO_x film thickness for the nanostructured 7 at% Rh containing GaRh alloy samples oxidized in 1×10^{-6} mbar O_2 for different times.

Oxidation time	GaO_x/Ga ratio in Ga 2p	GaO_x thickness (Å)
0 min	0.06±0.1	0.5±0.5
10 min	0.3±0.2	2.3±1
30 min	1.0±0.2	5.3±1
60 min	1.5±0.1	6.5±1
240 min	1.8±0.1	7.3±1

Table S2. GaO_x/metallic Ga ratio of the respective spectral contributions to the Ga 3d core level and calculated GaO_x film thickness for the nanostructured 7 at% Rh containing GaRh alloy samples oxidized in 1×10^{-6} mbar O_2 for different times.

Oxidation time	GaO_x/Ga ratio in Ga 3d	GaO_x thickness (Å)
0 min	0±0.1	0±1
10 min	0.08±0.05	2.9±1
30 min	0.20±0.03	6.5±1
60 min	0.29±0.03	8.8±1
240 min	0.30±0.03	9.0±1

Table S3. Ga $3d_{5/2}$ and Rh $3d_{5/2}$ peak areas (as determined in Fig. S5 and S7) and XPS derived Rh content of the GaRh alloys oxidized in 1×10^{-6} mbar O_2 for different times (0-240 min). The IMFP value is calculated by the TPP-2M formula.[2-4] Photoionization cross section (σ) is taken from the tabulated values from Trzhaskovskaya, Nefedov, and Yarzhemski.[5-6]

Oxidation time	Core level	Peak area (a.u.)	Cross-section (a.u.)	IMFP (Å)	Rh/Ga ratio	Rh concentration (at%)
before oxidation (0 min)	Ga $3d_{5/2}$	10794.8	1.61	20.9	0.072±0.005	6.7±0.5
	Rh $3d_{5/2}$	7583.6	19.2	17.2		
10 min	Ga $3d_{5/2}$	10650.2	1.61	20.9	0.079±0.005	7.2±0.5
	Rh $3d_{5/2}$	6618.2	19.2	17.2		
30 min	Ga $3d_{5/2}$	9731.1	1.61	20.9	0.073±0.005	6.7±0.5
	Rh $3d_{5/2}$	3393.8	19.2	17.2		
60 min	Ga $3d_{5/2}$	9651.2	1.61	20.9	0.056±0.005	5.3±0.5
	Rh $3d_{5/2}$	5277.9	19.2	17.2		
240 min	Ga $3d_{5/2}$	9553.4	1.61	20.9	0.058±0.005	5.4±0.5
	Rh $3d_{5/2}$	5450.9	19.2	17.2		

Table S4. Ga $2p_{3/2}$ and Rh $3d_{5/2}$ peak areas (as determined in Fig. S6 and S7) and XPS derived Rh content of the GaRh alloys oxidized in 1×10^{-6} mbar O_2 for different times (0-240 min). The IMFP value is calculated by the TPP-2M formula.[2-4] Photoionization cross section (σ) is taken from the tabulated values from Trzhaskovskaya, Nefedov, and Yarzhemski.[5-6]

Oxidation time	Core level	Peak area (a.u.)	Cross-section (a.u.)	IMFP (Å)	Rh/Ga ratio	Rh concentration (at%)
before oxidation (0 min)	Ga $2p_{3/2}$	240785.4	45.7	5.3	0.023±0.005	2.2±1
	Rh $3d_{5/2}$	7583.6	19.2	17.2		
10 min	Ga $2p_{3/2}$	243891.6	45.7	5.3	0.025±0.005	2.4±1
	Rh $3d_{5/2}$	6618.2	19.2	17.2		
30 min	Ga $2p_{3/2}$	211918.4	45.7	5.3	0.024±0.005	2.3±1
	Rh $3d_{5/2}$	3393.8	19.2	17.2		
60 min	Ga $2p_{3/2}$	198068.4	45.7	5.3	0.020±0.005	1.9±1
	Rh $3d_{5/2}$	5277.9	19.2	17.2		
240 min	Ga $2p_{3/2}$	192251.3	45.7	5.3	0.021±0.005	2.0±1
	Rh $3d_{5/2}$	5450.9	19.2	17.2		

Table S5. GaO_x/metallic Ga ratio of the respective spectral contributions to the Ga $2p_{3/2}$ core level and calculated GaO_x film thickness for the nanostructured 7 at% Rh containing oxidized GaRh alloy samples annealing at different temperatures.

Temperature	GaO_x/Ga ratio in Ga 2p	GaO_x thickness (Å)
30 °C	1.78±0.1	7.3±1
300 °C	1.77±0.1	7.3±1
400 °C	1.57±0.1	6.8±1
600 °C	0.38±0.1	2.6±0.5
650 °C	0.27±0.1	2.0±0.5

Table S6. Ga $2p_{3/2}$ and Rh $3d_{5/2}$ peak areas (as determined in Fig. S9 and S11) and XPS derived Rh content of the GaRh alloys annealing in ≦ 1×10^{-8} mbar condition for different temperatures (30-650 °C). The IMFP value is calculated by the TPP-2M formula.[2-4] Photoionization cross section (σ) is taken from the tabulated values from Trzhaskovskaya, Nefedov, and Yarzhemski.[5-6]

Oxidation time	Core level	Peak area (a.u.)	Cross-section (a.u.)	IMFP (Å)	Rh/Ga ratio	Rh concentration (at%)
before annealing (30 °C)	Ga $2p_{3/2}$	192251.3	45.7	5.3	0.020±0.005	2.0±1
	Rh $3d_{5/2}$	5450.9	19.2	17.2		
300 °C	Ga $2p_{3/2}$	196546.3	45.7	5.3	0.018±0.005	1.8±1
	Rh $3d_{5/2}$	4939.1	19.2	17.2		
400 °C	Ga $2p_{3/2}$	212906.5	45.7	5.3	0.018±0.005	1.7±1
	Rh $3d_{5/2}$	5152.8	19.2	17.2		
600 °C	Ga $2p_{3/2}$	231649.2	45.7	5.3	0.013±0.005	1.3±1
	Rh $3d_{5/2}$	3930.4	19.2	17.2		
650 °C	Ga $2p_{3/2}$	217850.5	45.7	5.3	0.012±0.005	1.2±1
	Rh $3d_{5/2}$	3548.1	19.2	17.2		

References:

1. Hsieh, T.-E.; Frisch, J.; Wilks, R. G.; Bär, M., Unravelling the Surface Oxidation-Induced Evolution of the Electronic Structure of Gallium. *Appl. Mater. Interfaces* **2023,** *15*, 47725–47732.
2. Shinotsuka, H.; Tanuma, S.; Powell, C. J.; Penn, D. R., Calculations of Electron Inelastic Mean Free Paths. XII. Data for 42 Inorganic Compounds over The 50 eV to 200 keV Range with The Full Penn Algorithm. *Surf. Interface Anal.* **2019,** *51*, 427-457.
3. Shinotsuka, H.; Da, B.; Tanuma, S.; Yoshikawa, H.; Powell, C. J.; Penn, D. R., Calculations of Electron Inelastic Mean Free Paths. XI. Data for Liquid Water for Energies from 50 eV to 30 keV. *Surf. Interface Anal.* **2017,** *49*, 238-252.
4. Shinotsuka, H.; Tanuma, S.; Powell, C. J.; Penn, D. R., Calculations of Electron Inelastic Mean Free Paths. X. Data for 41 Elemental Solids over The 50 eV to 200 keV Range with The Relativistic Full Penn Algorithm. *Surf. Interface Anal.* **2015,** *47*, 1132-1132.
5. Trzhaskovskaya, M. B.; Nikulin, V. K.; Nefedov, V. I.; Yarzhemsky, V. G., Influence of Nondipolar Effects on The Photoelectron Angular Distribution upon Photoionization of 2p and 3d Atomic Shells. *Opt. Spectrosc.* **2004,** *96*, 765-773.
6. Trzhaskovskaya, M. B.; Nefedov, V. I.; Yarzhemsky, V. G., Photoelectron Angular Distribution Parameters for Elements Z=1 to Z=54 in the Photoelectron Energy Range 100–5000 eV. *At. Data Nucl. Data Tables* **2001,** *77*, 97-159.
7. Wolfgang, W.; Werner, S.; Cedric, P.; Justin, G., Simulation of Electron Spectra for Surface Analysis (SESSA)Version 2.2 User's Guide. Natl Std. Ref. Data Series (NIST NSRDS), National Institute of Standards and Technology, Gaithersburg, MD: 2021.
8. Seah, M. P.; Dench, W. A., Quantitative Electron Spectroscopy of Surfaces: A Standard Data Base for Electron Inelastic Mean Free Paths in Solids. *Surf. Interface Anal.* **1979,** *1*, 2-11.
9. Jeurgens, L. P. H.; Sloof, W. G.; Tichelaar, F. D.; Mittemeijer, E. J., Composition and Chemical State of the Ions of Aluminium-Oxide Films Formed by Thermal Oxidation of Aluminium. *Surf. Sci.* **2002,** *506*, 313-332.
10. Wittkämper, H.; Maisel, S.; Wu, M.; Frisch, J.; Wilks, R. G.; Grabau, M.; Spiecker, E.; Bär, M.; Görling, A.; Steinrück, H.-P.; Papp, C., Oxidation Induced Restructuring of Rh–Ga SCALMS Model Catalyst Systems. *J. Chem. Phys.* **2020,** *153*, 104702.
